# A proteomic screen of Ty1 integrase partners identifies the protein kinase CK2 as a regulator of Ty1 retrotransposition

**DOI:** 10.1186/s13100-022-00284-0

**Published:** 2022-11-18

**Authors:** Anastasia Barkova, Indranil Adhya, Christine Conesa, Amna Asif-Laidin, Amandine Bonnet, Elise Rabut, Carine Chagneau, Pascale Lesage, Joël Acker

**Affiliations:** 1 Université Paris Cité, CNRS, Inserm, Génomes biologie cellulaire et thérapeutiques, F-75010 Paris, France; 2 Université Paris-Saclay, CEA, CNRS, Institute for Integrative Biology of the Cell (I2BC), 91198 Gif-sur-Yvette, France

**Keywords:** Ty1, Integrase, Proteomic, CK2 protein kinase, Phosphorylation, Retrotransposition, *Saccharomyces cerevisiae*

## Abstract

**Background:**

Transposable elements are ubiquitous and play a fundamental role in shaping genomes during evolution. Since excessive transposition can be mutagenic, mechanisms exist in the cells to keep these mobile elements under control. Although many cellular factors regulating the mobility of the retrovirus-like transposon Ty1 in *Saccharomyces cerevisiae* have been identified in genetic screens, only very few of them interact physically with Ty1 integrase (IN).

**Results:**

Here, we perform a proteomic screen to establish Ty1 IN interactome. Among the 265 potential interacting partners, we focus our study on the conserved CK2 kinase. We confirm the interaction between IN and CK2, demonstrate that IN is a substrate of CK2 in vitro and identify the modified residues. We find that Ty1 IN is phosphorylated in vivo and that these modifications are dependent in part on CK2. No significant change in Ty1 retromobility could be observed when we introduce phospho-ablative mutations that prevent IN phosphorylation by CK2 in vitro*.* However, the absence of CK2 holoenzyme results in a strong stimulation of Ty1 retrotransposition, characterized by an increase in Ty1 mRNA and protein levels and a high accumulation of cDNA.

**Conclusion:**

Our study shows that Ty1 IN is phosphorylated, as observed for retroviral INs and highlights an important role of CK2 in the regulation of Ty1 retrotransposition. In addition, the proteomic approach enabled the identification of many new Ty1 IN interacting partners, whose potential role in the control of Ty1 mobility will be interesting to study.

**Supplementary Information:**

The online version contains supplementary material available at 10.1186/s13100-022-00284-0.

## Introduction

**Fig. 1:**
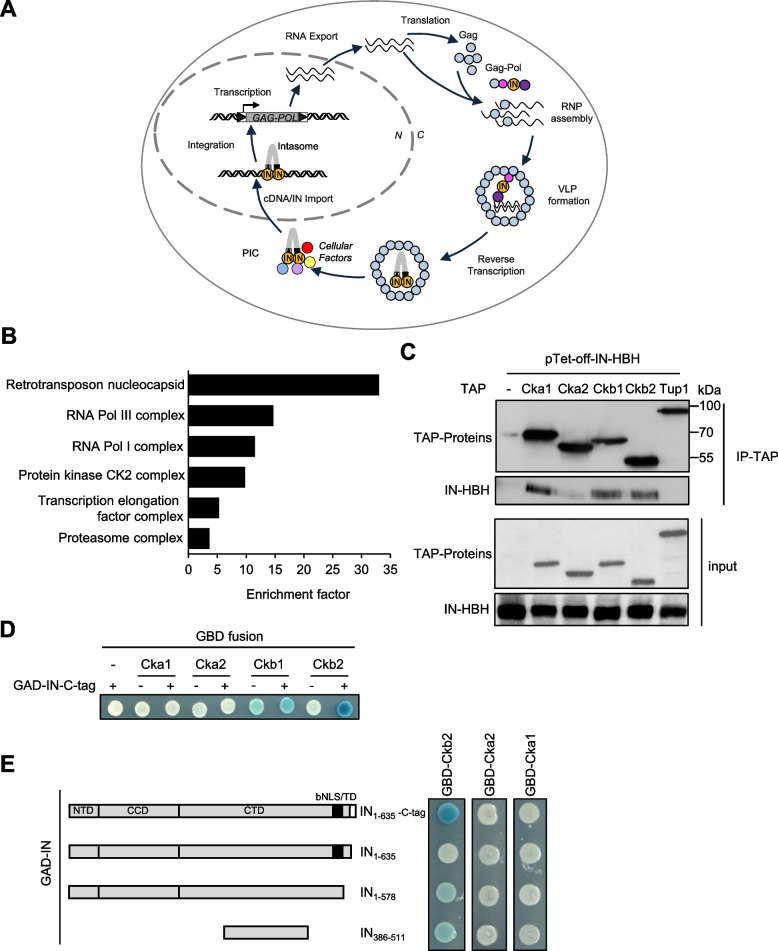
Large-scale purification of Ty1 integrase binding partners. **A** Ty1 replication cycle. A Ty1 element (grey bar with black triangles at both ends) is transcribed into RNAs that are exported to the cytoplasm and subsequently translated into Gag and Gag-Pol proteins that associate with Ty1 RNA to form Ty1 ribonucleoparticles called retrosomes. The retrosome is the assembly-site for virus-like particles (VLPs) that contains tRNAiMet and a dimer of Ty1 RNA. Within the VLP, Gag and Pol proteins are cleaved by the protease (PR, pink ball) to form mature Gag (blue ball), PR, integrase (IN, orange ball) and reverse transcriptase (RT, purple ball) proteins, and Ty1 RNA is reverse transcribed into cDNA by RT using tRNAiMet as a primer. Then, IN binds to Ty1 cDNA to form the pre-integration complex that contains additional host (yellow and red balls) and retroelement proteins (blue and purple balls) and is imported into the nucleus, where it catalyzes the integration of Ty1 cDNA into the yeast genome. **B** Functional classification of the Ty1 IN partners based on Gene Ontology (GO) cellular component. GOrilla algorithm [4] was used to retrieve the enriched GO terms within the complete set of proteins identified by TChAP. Only GO hits with an FDR-corrected q-value ≤0.01 were considered statistically significant in the final analysis. Numbers on X-axis indicate the enrichment in each identified GO cluster. Full list of the enriched GO terms is available in Table S3. **C** CK2 is associated with ectopic IN in vivo. Whole cell extracts from yeast cells expressing WT or TAP-tagged CK2 subunits and expressing or not (−) ectopic IN-HBH from a pTet-off promoter were immunoprecipitated with IgG beads. Proteins were analyzed by Western blot using anti-strep (IN-HBH) or anti-TAP antibodies (CK2). TAP-Tup1 is used as a negative control. Molecular weights are indicated. **D** Two-hybrid interactions between CK2 subunits and IN. Each CK2 subunit was fused to GAL4 binding domain (GBD) and IN-C-tag to GAL4 activating domain (GAD). GBD or GAD empty vector (−) serves as control. Cells were plated on selective medium and incubated for 2 days. Activation of the *LacZ* reporter gene was monitored by staining in the presence of X-Gal. **E** Two-hybrid interaction between GBD-Cka1, −Cka2, −Ckb2 and different IN regions, as depicted on the left panel, fused to GAD. Cells were plated on selective medium and incubated for 2 days. Activation of the *LacZ* reporter gene was monitored by staining in the presence of X-Gal. The black square corresponds to the bNLS which contains the targeting domain (TD)

Transposable elements are mobile genetic elements that propagate in genomes. They represent a major source of genetic variation and innovation and as such contribute to species evolution [1]. Long-terminal repeat (LTR)-retrotransposons, one of the major classes of retrotransposons, are evolutionary related to infectious retroviruses [2]. LTR-retrotransposons and retroviruses are referred to as LTR-retroelements. The replication of LTR-retrotransposons is a complex process, in which genomic RNA is transcribed from a chromosomal copy and serves as a template for both protein and complementary DNA (cDNA) synthesis (Fig. 1A). The precursor Gag and Gag-Pol proteins are processed into mature Gag, protease (PR), integrase (IN) and reverse transcriptase (RT) proteins within cytoplasmic virus-like particles (VLP) made of structural Gag proteins. After reverse transcription of the genomic RNA in the VLPs, the cDNA is associated with IN, that together with retroelement and cellular proteins, form a pre-integration complex (PIC) [3], which transits into the nucleus, where IN catalyzes cDNA integration into the host genome.

Crystallographic studies have characterized in vitro the structure of the minimal functional complex of cDNA and IN, called intasome (Fig. 1A), from several retroviruses and revealed a globally conserved organization, with a multimer of INs assembled on cDNA ends [5–8]. The composition and architecture of the PIC of LTR-retrotransposons remain largely unknown, not only because of its low abundance in the cell but also because its composition might change during LTR-retrotransposon replication. Among the cellular proteins known to interact with INs, some of them, called tethering factors, direct the integration to specific sites of the genomes [9]. Tethering factors have been identified for the HIV-1 and MLV retroviruses [10–13], the Tf1 LTR-retrotransposon of *Schizosaccharomyces pombe* [14, 15] as well as the Ty1, Ty3 and Ty5 LTR-retrotransposons of *Saccharomyces cerevisiae* [16–18]. In the case of HIV-1, IN also interacts with the protein kinase GCN2, the E3 ubiquitin ligase TRIM33 and SUMO proteins that modify IN post-translationally to modulate its activity, stability or interaction with cofactors [19–22], as well as with the chromatin remodeler FACT, which helps the association of the intasome with the targeted nucleosome [23]. Therefore, IN exhibits multiple interactions with cellular proteins of various functions highlighting the central role of IN in the replication of LTR-retroelements.

Ty1 is the most abundant and active LTR-retrotransposon in *S. cerevisiae*. Ty1 IN is a 71-kDa protein of 635 amino acid residues, produced from a rather complex semi-ordered Gag-Pol proteolytic cleavage pathway [24]. Ty1 IN harbours the conserved three domain structure of retroviral integrases: the N-terminal oligomerization domain (NTD) containing a conserved HHCC zinc-binding motif, the central catalytic core domain (CCD) with the invariant DD_35_E motif and the less conserved C-terminal domain (CTD)*,* which, in the case of Ty1, is particularly large and ends with an intrinsically disordered region [25]. The last 115 C-terminal residues of Ty1 IN are involved in multiple interactions important for Ty1 replication cycle. This region interacts *in cis* with Ty1 reverse transcriptase to stimulate its activity in vitro and in vivo [26, 27]. The IN C-terminus also contains a bipartite nuclear localization signal (bNLS), which is recognized by the importin-α that triggers Ty1 PIC import into the nucleus [28–30]. A stretch of residues in the linker sequence of the bNLS, named the targeting domain, interacts with the AC40 subunit of RNA polymerase III (Pol III), an interaction determinant for Ty1 PIC recruitment to Pol III-transcribed genes and Ty1 preferential integration upstream of these genes [18, 31]. Ty1 IN also interacts in vivo with the Esp1 protease, which is involved in mitotic sister chromatid separation and enhances Ty1 replication [32]. Besides, a plethora of cellular factors controlling positively or negatively Ty1 replication have been identified in genetic screens [33–37] but very few cellular proteins interacting directly with IN have been identified so far [38].

Here we report a proteomic screen by chromatin affinity purification that allowed us to build a large repertoire of Ty1 IN cofactors, and focus on one of them, the CK2 serine/threonine protein kinase. CK2 is highly conserved from budding yeast to mammalian cells [39]. CK2 is ubiquitously expressed, constitutively active and has been implicated in various cellular processes related to cell growth, cell cycle progression, viability, transcription, histone dynamics, signalling, proliferation and viral replication [40, 41]. We confirm the interaction between Ty1 IN and the CK2 holoenzyme, demonstrate that IN is a substrate of CK2 in vitro and identify the amino acids phosphorylated in vitro. IN is also highly phosphorylated in vivo, this phosphorylation being in part dependent on CK2. We further show that CK2 strongly represses Ty1 retrotransposition without finding a role for CK2-dependent IN phosphorylation in this repression. Instead, we observe an increase in the amounts of Ty1 mRNA, proteins and cDNA in the absence of CK2.

## Results

### Identification of Ty1 integrase binding partners

To identify yeast proteins associated with Ty1 IN that could regulate Ty1 replication, we adapted the tandem chromatin affinity purification procedure after in vivo cross-link (TChAP), which we developed previously [42] (Fig. S1A). IN protein was expressed with a C-terminal histidine-biotin-histidine (HBH) tag [43], allowing ectopic IN-HBH purification under denaturing conditions. Proteins that co-purified with IN-HBH were compared to those detected in the control yeast strain expressing untagged IN, and IN-HBH specific partners were sorted according to the settings of TChAP selection criteria [42]. Ultimately, we identified a set of 265 IN binding proteins (Table S1), which expands the number of candidates previously described [38]. Many of these proteins were nuclear. Their recovery was consistent with IN function in Ty1 integration into the nuclear genome and with the TChAP chromatin purification step. In addition, several peptides mapped to Gag, PR, IN and RT protein sequences from Ty1 and Ty2 retrotransposons, which are closely related, suggesting that ectopic IN-HBH interacts with endogenous Ty1 or Ty2 proteins (Table S2).

To get insights into the cellular activity associated with IN binding partners, the proteins identified by TChAP were classified using Gene ontology (GO) enrichment analysis and the GOrilla visualization tool (Fig. 1B, Table S3A-D) and revealed a limited number of highly enriched biological processes (*q*-value < 4.3 E-6), including transposition due to the presence of Ty1 and Ty2 peptides, but also RNA Pol I and Pol III transcription (Fig. S1B). GO cellular components analysis revealed a specific enrichment of RNA Pol I and Pol III polymerases, with the identification of many of their subunits. These data are consistent with previous studies showing a physical interaction between IN and both RNA polymerases [31, 38] (Fig. 1B). In particular, their common subunit AC40 was overrepresented and had the largest protein coverage in the TChAP experiment (Table S1), a finding consistent with the previously described IN-AC40 interaction that targets Ty1 integration upstream of Pol III-transcribed genes [18, 31]. Other enriched GO biological processes were related to chromatin dynamics and histone modifications, suggesting that IN may interact with factors modulating chromatin state and content (Fig. S1B and Table S3A-B). IN was also associated with other evolutionary conserved transcription complexes, including PAF1 (Polymerase-Associated Factor 1) and FACT (FAcilitates Chromatin Transcription) (Table S3C-D and Fig. 1B). Several subunits of PAF1 were previously shown to restrict Ty1 retrotransposition, especially at coding genes [36], while FACT interacts with HIV-1 IN and stimulates HIV-1 infectivity and integration [23]. The crosslinking step performed during the TChAP may have helped to identify the association between IN and chromatin and transcriptional complexes.

Proteasome components were also found in our GO term analyses suggesting that IN may be regulated by proteasome-dependent degradation or that the proteasome degrades overexpressed IN-HBH. Finally, the four subunits of the serine/threonine/tyrosine protein kinase CK2 were identified (Table S1). The Ckb2 subunit was previously identified in a proteomic screen for Ty1 IN cofactors [38] and in genetic screens for Ty1 and Ty3 repressors [36, 44] but its role in Ty replication was not characterized.

In conclusion, we identified by TChAP a large number of proteins that are good candidates to interact with IN in vivo and regulate Ty1 replication. We decided to further explore the role of CK2 in Ty1 retrotransposition because the kinase i) is present at tRNA genes [45, 46], which are primary targets for Ty1 integration [47, 48], ii) phosphorylates several components of the Pol III machinery and iii) positively regulates Pol III transcription [45, 49, 50], suggesting that CK2 could interact with IN and control Ty1 integration at tRNA genes.

### The CK2 holoenzyme interacts with Ty1 integrase in vivo

CK2 mainly exists in *S. cerevisiae* as a tetramer composed of two regulatory subunits, Ckb1 and Ckb2, and two catalytic subunits, Cka1 and Cka2 either as homo or hetero-dimer [51] but free Cka2 has also been detected [52]. No single CK2 subunit is essential. Only the combined absence of the catalytic subunits Cka1 and Cka2 is lethal to the cell [53]. To validate the association between IN and CK2, we performed co-immunoprecipitation (co-IP) analyses. IN-HBH was expressed in a WT strain or in different strains, each expressing a TAP-tag version of Cka1, Cka2, Ckb1 or Ckb2 subunits or Tup1 as a negative control. IN-HBH co-purified with each of the TAP-tagged CK2 subunits immunoprecipitated from exponentially growing cells, but not with either Tup1-TAP or a control protein extract prepared from the WT strain lacking TAP-tag proteins (Fig. 1C). These data strongly suggest that Ty1 IN is associated with CK2 holoenzymes in vivo.

To determine which CK2 subunit interacts with IN, a two-hybrid assay using Ty1 IN as bait and the four CK2 subunits as preys was conducted and revealed an interaction between Ty1 IN and the Ckb2 regulatory subunit (Fig. 1D). This interaction could only be detected when the full-length Ty1 IN carried a C-tag at its C-terminus, suggesting that this tag could interact with Ckb2 or promotes a structural conformation of Ty1 IN that allows its interaction with Ckb2 (Fig. 1E). Additional experiments showed that the interaction with Ckb2 occurred in the absence of the bNLS, the targeting domain and the C-tag (Fig. 1E, GAD-IN_1–578_) and that Ty1 IN amino acids 386 to 511 were sufficient for the interaction (Fig. 1E, GAD-IN_386–511_). In contrast, no physical association could be detected between IN and any of the catalytic subunits. We could not conclude for Ckb1 as GBD-Ckb1 self-activated the expression of the reporter gene. Together, these results point to a physical interaction between IN and CK2 holoenzymes that likely depends on the binding of Ckb2 to a sequence located in the C-terminal domain (CTD) of IN.

To investigate whether CK2 also interacts with IN expressed from a functional Ty1 element, we immunoprecipitated CK2 in yeast cells expressing TAP-tagged Cka2 or Ckb1 proteins and a Ty1 element under the control of a Tet-off promoter to increase cellular IN protein levels (Fig. S2). IN was associated with the two TAP-tagged CK2 subunits but not with TAP-YKR011C as a negative control and was not detected in the absence of TAP-proteins. However, the co-IP was difficult to detect presumably because of the low level of IN processed from Gag-Pol compared to that of IN-HBH (Fig. 1C).

Altogether, these data indicate that in yeast cells, the protein kinase CK2 interacts with ectopic Ty1 IN expressed alone or upon maturation of Ty1 Gag-Pol polyprotein.

### CK2 phosphorylates Ty1 integrase in vitro

**Fig. 2:**
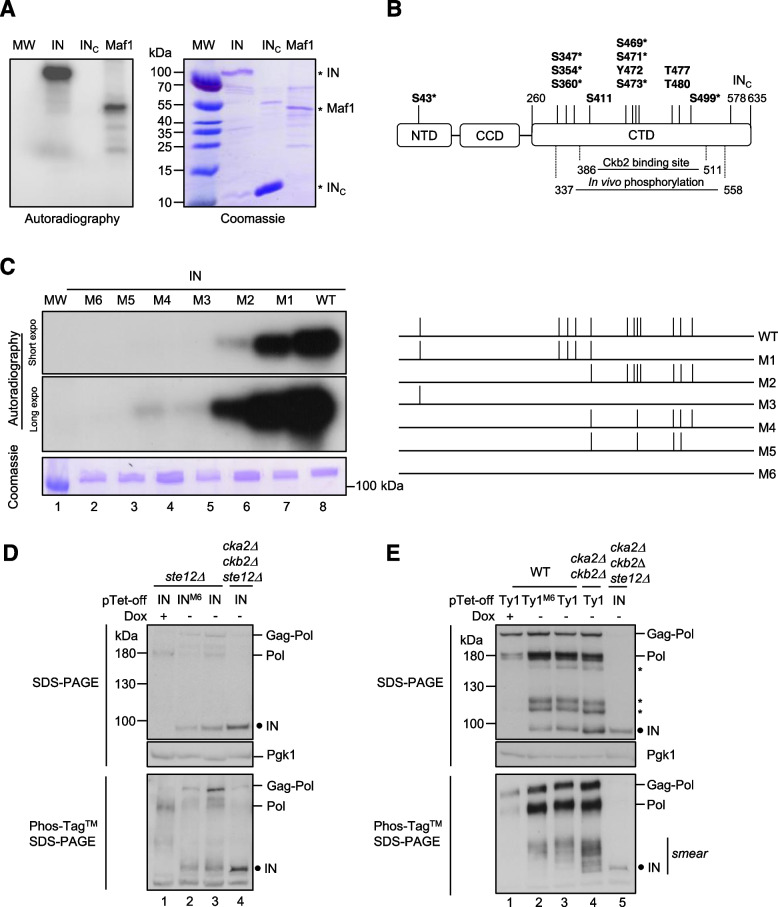
CK2 phosphorylates Ty1 integrase in vitro and is involved in its phosphorylation in vivo. **A** Full-length IN (200 ng), IN_578–635_ (IN_C_) (500 ng) or Maf1 (200 ng) expressed in *E. coli* were subjected to in vitro radioactive phosphorylation assays with purified yeast CK2 holoenzyme. Incorporation of γ^32^P was detected by autoradiography (left panel), the loading of the recombinant proteins was analyzed by Coomassie blue staining (right panel). MW: protein marker. Full length IN, IN_C_ and Maf1 are indicated with an asterisk (*). **B** Mapping of CK2 phosphorylation sites in Ty1 IN. A schematic representation of IN with the N-terminal (NTD), the catalytic core (CCD) and the C-terminal domain (CTD) is depicted [25]. Starting and ending residues for the CTD, the IN_C_ sequence and Ckb2 binding site are indicated. All amino acids within a CK2 consensus site listed in Table S4A (Netphos 3.1 analysis) are indicated with an asterisk (*). The 12 amino acids potentially phosphorylated by CK2 in the endogenous IN, based on Phosphogrid database, are shown in bold (Table S4B). **C** In vitro kinase assays of IN phosphomutants. *Left*: Autoradiography (incorporation of γ^32^P) and Coomassie blue staining of WT and mutant IN proteins purified from *E. coli* (200 ng). MW: protein marker. *Right*: Mutations preventing phosphorylation of specific residues of IN shown in panel B are indicated by the absence of a vertical line in the representation of IN mutants M1 to M6. **D** Phosphorylation of ectopically IN expressed alone is not dependent on CK2 in vivo. Total protein extracts from *ste12Δ* or *cka2Δ cbk2Δ ste12Δ* cells expressing WT or IN^M6^ (as indicated in panel C) from pTet-off-IN plasmids were separated by SDS-PAGE in the absence (upper panel) or the presence of Phos-Tag™ (lower panel). Pgk1 is a loading control. The presence of IN and Pol intermediates produced from endogenous Ty1 elements was detected by Western blot using rabbit anti-IN polyclonal antibodies. The black circle corresponds to the dephosphorylated form of IN. **E** Phosphorylation of IN produced from an ectopic Ty1 element depends in part on CK2 in vivo*.* Western blot analysis as described in panel D of total protein extracts prepared from cells expressing ectopic Ty1 or Ty1^M6^ mutant from pTet-off plasmids as indicated. Asterisks indicate Pol intermediates from endogenous and ectopic Ty1 elements. IN expressed from pTet-off-IN was used as a control. In Phos-Tag™ SDS-PAGE, the smear above IN indicates the presence of several forms of phosphorylated IN or Pol intermediates. A similar profile was reproduced with cell extracts from 3 independent experiments

The interaction between Ty1 IN and CK2 observed in vivo suggested that CK2 could directly phosphorylate the integrase. Ty1 IN (635 amino acids) harbours as much as 78 serines and 52 threonines that could be phosphorylated. A minimal consensus sequence for CK2 phosphorylation (S/T–X–X–D/E) has been described but deviations from the consensus have already been observed [40]. To determine whether Ty1 IN is a substrate of CK2, we performed in vitro kinase assays with recombinant Ty1 IN produced in *E. coli* in the presence of either yeast CK2 holoenzymes or commercial recombinant human rCK2. With both kinases, a radiolabelled signal was detected in the presence of γ^32^P-ATP for Maf1, a known substrate of CK2 [45], as well as for the full-length IN. This confirms that Ty1 IN is a substrate of CK2 in vitro. In agreement with our two-hybrid assay, a C-terminal fragment of IN (amino acids 578–635), which harbours potential phosphorylation sites, encompasses the bipartite NLS and the Ty1 targeting domain but does not interact with Ckb2, did not show radioactive incorporation, indicating that this region of Ty1 IN is not a substrate for CK2 (Fig. 2A, Fig. S3A and Fig. 1E).

To identify the amino acids phosphorylated by CK2, we performed a mass spectrometry (MS) analysis of in vitro phosphorylated Ty1 IN and identified 12 amino acids, including 9 serines, 2 threonines and one tyrosine (Fig. 2B and Table S4B*).* To confirm the role of these residues in the phosphorylation by CK2, we performed in vitro kinase assays using similar amounts of purified IN variants, where the candidate serine or threonine residues were changed into alanine and the tyrosine (Y472) residue into phenylalanine to prevent their phosphorylation (Fig. 2C, left and right panels). No radioactive signal could be detected when all 12 amino acids were mutated simultaneously (Fig. 2C, mutant M6). The same loss of signal was observed when we mutated together the 8 amino acids present in a CK2 consensus (Netphos analysis, Table S4A), indicating that these residues are the only ones efficiently phosphorylated by CK2 in vitro (Fig. 2C, mutant M5). MS can detect a minimal number of phosphorylated molecules that would not be sufficient to provide a radioactive signal in vitro. The high sensitivity of MS could explain the discrepancy between MS data and in vitro phosphorylation assays. Thus, only residues that were also phosphorylated in vitro were considered as convincing substrates of CK2.

In comparison, the residual radioactive signal observed in M3 and M4 mutants revealed that S499 and S43 were phosphorylated by CK2 in vitro. Finally, the reduction in the autoradiographic signal of the M1 and M2 mutants relative to IN indicated that both serine clusters (S347, S354 and S360 or S469, S471 and S473, respectively) were targeted by CK2 in vitro with no clear definition of the substrate serines.

Together, these results indicate that S43, S499 and one or more serines in the S347-S354-S360 and S469-S471-S473 clusters account for the majority, if not all, of CK2-mediated IN phosphorylation in vitro.

### IN is phosphorylated during Ty1 replication

To determine whether IN was also phosphorylated in a cellular context, we purified ectopic IN-HBH expressed in yeast cells and analyzed its in vivo phosphorylation profile by MS. All in vivo phosphorylated amino acids were located within or near IN residues 386 to 511 that interact with Ckb2 in two-hybrid experiments (Fig. 1E and Table S4B). The data also indicated that all but one amino acid identified as CK2 targets in vitro were also phosphorylated in vivo. The only exception concerned residue S43, located in a region that was absent from the peptide sequences detected by MS, hampering any conclusion about its modification in vivo. Ten additional phosphopeptides were detected only in the cellular context (Table S4B). All the amino acids phosphorylated in ectopically expressed IN, both in vivo and in vitro, were also present in the PhosphoGRID database, which compiles experimentally verified endogenous protein phosphorylation sites in *S. cerevisiae* (Table S4B and http://www.phosphogrid.org/ [54]), indicating that endogenous and ectopic INs harbour the same modifications. Finally, the comparison of the phosphorylation sites identified by the different approaches (in vitro, in vivo and of endogenous IN) with the consensus sequence for phosphorylation by CK2 yielded a list of 12 amino acids potentially phosphorylated by CK2 in the endogenous IN (S43, S347, S354, S360, S411, S469, S471, Y472, S473, T477, T480 and S499).

To assess the contribution of CK2 to IN phosphorylation in vivo, we expressed IN alone from a pTet-off inducible vector in *ste12∆* and *cka2∆ ckb2∆ ste12∆* mutant cells. Of note, *STE12* deletion prevents Ty1 endogenous expression [55] and the *cka2∆ ckb2∆* double mutation affects CK2 holoenzyme formation [51]. On a conventional SDS-PAGE gel, ectopically expressed IN was detected as a single band with similar mobility in *ste12∆* and *cka2Δ ckb2Δ ste12Δ* protein extracts (Fig. 2D, upper panel, compare lanes 3 and 4). In addition, the IN^M6^ protein, which harbours phospho-ablative mutations on the 12 amino acids potentially modified by CK2 in vivo*,* had the same migration pattern as IN in the *ste12∆* strain. The only noticeable difference between yeast extracts was higher IN amounts in the absence of Cka2 and Ckb2 (Fig. 2D, top panel, compare lanes 3 and 4), suggesting a putative role of CK2 on IN stability or expression from pTet-off. On Phos-tag™ SDS-PAGE, which allows a better separation of phosphorylated and non-phosphorylated proteins, we did not detect significant differences in migration between IN or IN^M6^ in *ste12∆* cells, and IN in *cka2∆ ckb2∆ ste12∆* cell extracts, which migrated mainly as a single discrete band (Fig. 2D, lower panel). These data indicate that ectopic IN produced in yeast cells is not significantly phosphorylated by CK2 in vivo under normal growth conditions.

We showed above that CK2 interacts with IN expressed from an ectopic Ty1 element (Fig. S2). Thus, we wondered whether IN produced during Ty1 replication might be a better substrate for CK2 in vivo. In yeast cells, IN is produced by successive cleavages of the precursor polyprotein Gag-Pol in Ty1 VLPs [24]. The Gag-Pol polyprotein has three distinct cleavage sites at the Gag/PR, PR/IN and IN/RT junctions. The Gag/PR site is cleaved first, but no further processing order was identified [24] and thus, in addition to the mature IN, various Gag-Pol intermediates including IN-RT and PR-IN could be detected by an antibody raised against IN (Fig. 2E, upper panel and [24]). To examine IN phosphorylation during Ty1 replication, Ty1 or Ty1^M6^ harbouring the same mutations in the IN sequence described in Fig. 2C, were expressed from a pTet-off promoter in WT and *cka2∆ ckb2∆* cells. The endogenous Gag-Pol and Pol precursors, but not the processed IN, could be observed when the expression of the ectopic Ty1 elements was repressed in the presence of doxycycline (Fig. 2E, lane 1 in both panels). In the absence of doxycycline, processed IN and additional IN precursors or intermediates were detected (Fig. 2E, lanes 2–4). On conventional SDS-PAGE, the mature INs expressed from Ty1 and Ty1^M6^ migrated as a single band, as observed when the two IN and IN^M6^ proteins were ectopically expressed alone (Fig. 2E and D, upper panel, lanes 2 and 3), and the migration profile of IN was similar in WT and *cka2∆ ckb2∆* cell extracts (Fig. 2E, upper panel, lanes 3 and 4). The sizes of the upper bands were consistent with Gag-Pol (200 kDa), Pol (160 kDa) and IN-RT (134 kDa), while the two bands above IN may correspond to Pol intermediates including PR-IN (92 kDa) (Fig. 2E, upper panel). As for IN expression alone, IN expressed from Ty1 was more abundant in the absence of Cka2 and Ckb2 (Fig. 2E, top panel, compare lanes 3 and 4 with Fig. 2D). The pattern observed on Phos-tag™ SDS-PAGE was different with the presence of multiple bands and smears in both WT and *cka2∆ ckb2∆* extracts expressing Ty1 or Ty1^M6^, which migrated slower than ectopic IN expressed alone from *cka2∆ ckb2∆ ste12∆* cell extracts (Fig. 2E, lower panel, compare lanes 2–4 with lane 5). This complex pattern reflects the presence of various phosphorylated mature and unprocessed INs. The faster mobility bands in *cka2∆ ckb2∆* cell extracts compared to wild-type extracts are consistent with a role for CK2 in IN phosphorylation during Ty1 replication (Fig. 2E, lower panel, compare lanes 3 and 4). However, the difference between Ty1 or Ty1^M6^ profiles in WT cell extracts was too small to infer that IN is a direct substrate of CK2 in vivo.

In conclusion, IN expressed from an ectopic Ty1 element is phosphorylated in vivo under normal growth conditions and these modifications are directly or indirectly dependent on CK2. Noteworthy, the detection of several distinct bands by Western blot, when IN is expressed from a Ty1 element, suggests that mature IN produced in vivo during Ty1 replication is a better substrate for kinases than IN expressed alone (Fig. 2E and D, compare lanes 2 and 3).

### The CK2 protein kinase inhibits Ty1 retrotransposition

The interaction between IN and CK2 suggested that the kinase could regulate Ty1 retrotransposition. The regulatory subunit Ckb2 was previously found to repress Ty1 retrotransposition in a genetic screen [36]. To investigate the role of CK2 in the control of Ty1 retrotransposition, we performed a quantitative retrotransposition assay, based on the presence of a Ty1-*his3AI* reporter, which is naturally integrated in the genome and confers histidine prototrophy to cells that have undergone a new integration event [56] (Fig. S4A).

**Fig. 3:**
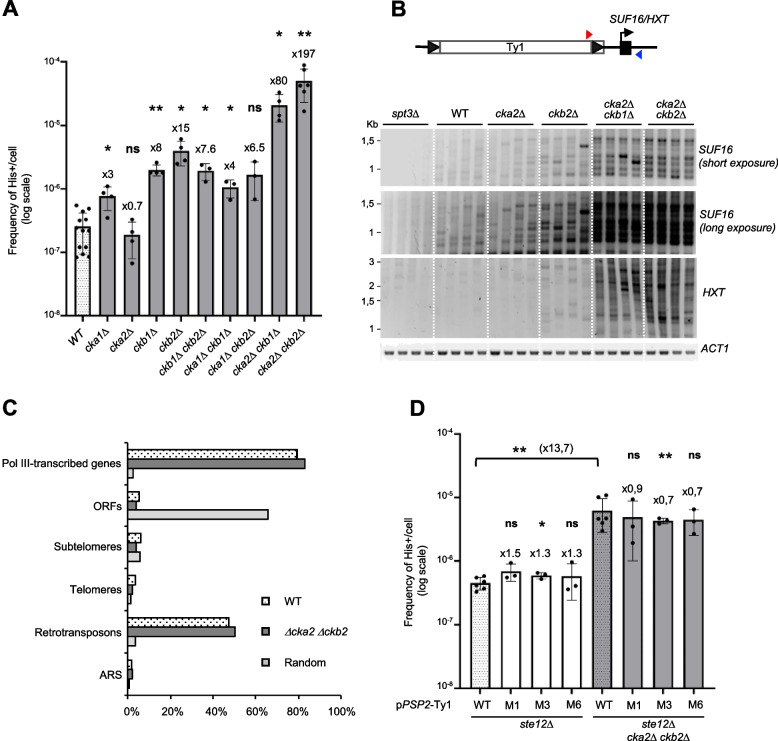
CK2 holoenzyme inhibits Ty1 retrotransposition. **A** Retrotransposition frequencies (log scale, mean ± SD, *n* ≥ 3) of a chromosomal Ty1-*his3AI* reporter in WT cells and non-essential mutants of the CK2 complex. Welch’s t-test with comparison to the WT strain: ns, not significant; *p < 0.05; **p < 0.01, ***p < 0.001. **B** Detection by PCR of de novo insertions of endogenous Ty1 elements upstream of the Pol III-transcribed gene *SUF16* and at the *HXT* subtelomeric loci in the indicated strains using a primer in Ty1 (red triangle) and a primer in the locus of interest (blue triangle). Total genomic DNA was extracted from His^+^ cells obtained from 4 independent cultures. Two different exposures are displayed for the *SUF16* locus to clearly show the increase in insertions in the *cka2∆ ckb2∆* mutant compared to the other strains. **C** Genome-wide Ty1-*HIS3* insertions. Percentage of insertions that occurred 1 kb-window upstream of Pol III-transcribed genes, in verified ORFs, subtelomeres, telomeres, retrotransposons, ARS and random insertions generated in silico*. ***D** Retrotransposition frequencies (log scale, mean ± SD, *n* ≥ 3) of a p*PSP2*-Ty1-his3AI reporter carried on a centromeric plasmid in the indicated cells. Welch’s t-test with comparison to the WT strain is indicated above each bar. Welch’s t-test with comparison between WT pPSP2-Ty1-his3AI in ste12∆ and ste12∆ cka2∆ ckb2∆ strains is indicated by a bracket. ns, not significant; *p

We measured retrotransposition frequencies in strains harbouring deletions of one or two CK2 subunits, except the *cka1∆ cka2∆* combination that is lethal [53]. The absence of either of the Cka1 or Cka2 catalytic subunits caused little or no increase in retrotransposition (Fig. 3A). In contrast, the absence of the Ckb1 or Ckb2 regulatory subunits elicited about the same increase, 8-fold to 15-fold respectively. No additive effect could be detected when both subunits were deleted. Both Ckb1 and Ckb2 are required for the formation of CK2 holoenzymes, while the catalytic subunits Cka1 and Cka2 are partially redundant within the complex [53]. These data strongly argue that it is primarily the CK2 holoenzymes that control Ty1 retrotransposition. The deletion of *CKA1* in the *ckb1∆* and *ckb2∆* mutants did not alter Ty1 retrotransposition, while the deletion of *CKA2* in these mutants further increased Ty1 retrotransposition up to 80-fold to nearly 200-fold, respectively (Fig. 3A). This strong derepression suggests that Cka2 might also participate to the restriction of Ty1 mobility in the absence of CK2 holoenzymes.

We next asked by which mechanism CK2 could repress Ty1 retrotransposition. Because post-translational modifications can affect the enzymatic activity of retroviral integrases [20, 57, 58], we first analyzed the catalytic activity of a phosphomimetic mutant of Ty1 IN, in which the eight amino acids phosphorylated in vitro by CK2 (Fig. 2C) were replaced by aspartic acid residues (M5D mutant) (Fig. S3B, upper panel). We performed an in vitro oligonucleotide integrase assay with short blunt-end double-stranded DNA probes of 30 bp, mimicking the end of the U3 region of Ty1 LTR, as previously described [25]. As shown in Fig. S3B, no significant difference could be detected between the strand transfer activity of WT and M5D mutant, which does not support a role for phosphorylation in the catalytic activity of IN.

Ty1 integrates primarily in a 1-kb window upstream of Pol III-transcribed genes but subtelomeres are secondary target sites when the interaction between Ty1 IN and RNA Pol III is abolished [18, 31]. Therefore, we examined whether CK2 could play a role in Ty1 integration site selection. We performed a dedicated PCR assay to compare the integration profile of endogenous Ty1 elements in WT and different CK2 mutants [59] (Fig. 3B). In this assay, proficient integration upstream of *SUF16*, a hotspot of Ty1 integration, is characterized by a ~ 70–base pair periodic banding pattern that corresponds to specific insertion events in nucleosomal DNA [18, 47, 48]. This pattern was markedly prominent in *cka2∆ ckb1∆* and *cka2∆ ckb2∆* mutants (Fig. 3B), which have the highest Ty1 integration frequency, and to a lesser extent in WT, *cka2∆* and *ckb2∆* cells, which have lower retrotransposition frequencies (Fig. 3A). As a control, no PCR signal was detected in an *spt3∆* mutant, which does not express endogenous Ty1 elements. These data indicate that *SUF16* remains a hotspot of Ty1 integration in CK2 mutants. Furthermore, when we analyzed Ty1 integration at four representative subtelomeric homologous genes (*HXT13*, *HXT15*, *HXT16* and *HXT17*), we also detected more de novo insertions in *cka2∆ ckb1∆* and *cka2∆ ckb2∆* mutants than in WT, *cka2∆* or *ckb2∆* cells (Fig. 3B). The band intensity at the *SUF16* and *HXT* loci clearly correlated with the retrotransposition frequencies in the mutants. Therefore, the increase in Ty1 integration frequency observed in CK2 mutants is associated with an increase in de novo insertion events at both primary and secondary Ty1 integration sites.

The above results indicate that CK2 does not participate in the recognition of Ty1 integration preferred sites but they do not exclude that CK2 could prevent Ty1 integration at other locations in the genome. Such a scenario has been reported for the retrotransposon Ty5 whose phosphorylation of the integrase targeting domain is essential for its interaction with the Sir4 protein and the integration of Ty5 into heterochromatin. In the absence of phosphorylation, Ty5 integration in the genome becomes random and can cause mutations [60]. To investigate the impact of CK2 on Ty1 genome-wide integration profile, libraries of His^+^ selected de novo Ty1 insertion events were generated in WT and *cka2∆ ckb2∆* cells expressing Ty1-*his3AI* elements from the *GAL1* promoter (Fig. S4B), and Ty1-*HIS3* de novo insertion sites were characterized by high-throughput sequencing, as done previously [18, 31]. Of note, the p*GAL1*-Ty1-*his3AI* element retrotransposes at a much higher frequency than the chromosomal Ty1-*his3AI* element and with similar frequencies in WT and *cka2∆ ckb2∆* cells (Fig. S4B and Fig. 3A). The calculated percentage of Ty1-*HIS3* insertions sequenced in representative features of the yeast genome indicated that de novo Ty1-*HIS3* insertions occurred mainly upstream of Pol III-transcribed genes and in retrotransposon sequences, most often present upstream of Pol III-transcribed genes, in both WT and *cka2∆ ckb2∆* cells (Fig. 3C). In addition, both strains displayed the same characteristic periodic pattern with two insertion sites per nucleosome in the three nucleosomes located upstream of Pol III-transcribed genes [47, 48] (Fig. S4C). Together, these data confirm that CK2 mutants do not alter Ty1 integration targeting at Pol III-transcribed genes. We did not identify any enrichment of de novo Ty1-*HIS3* insertions in different chromosome features (ORFs, subtelomeres, telomeres or ARS) in the *cka2∆ ckb2∆* cells compared to WT cells (Fig. 3C). Therefore, the increase in Ty1 insertions observed by PCR at the *HXT* subtelomeric loci is most likely a mere consequence of increased retrotransposition frequency and not of a change in Ty1 integration preferences.

The previous results support that CK2 does not control IN activity or its recognition of the integration site. To assess whether the repression by CK2 requires IN phosphorylation, we introduced the phospho-ablative mutations that prevent IN phosphorylation by CK2 in vitro (Fig. 2C, M1, M2 and M6 mutants) into a Ty1-*his3AI* reporter element, expressed from the TATA-less *PSP2* promoter, in place of the U3 promoter region of Ty1 LTR [61] (Fig. S4A). The *PSP2* promoter has a relatively weak activity [61] such that in WT cells the retrotransposition frequencies of Ty1-*his3AI* reporters expressed from the Ty1 promoter or from the *PSP2* promoter were of the same order of magnitude (Compare WT in Fig. 3A and Fig. 3D), thus approaching physiological retrotransposition conditions. To avoid possible trans-complementation by endogenous Ty1 elements [62], we expressed the p*PSP2*-Ty1-*his3AI* reporters in the absence of the Ste12 activator of Ty1 transcription [55]. The deletion of *STE12* reduced the levels of endogenous Ty1 mRNA by 2.5-fold, as expected, but not those of p*PSP2*-Ty1-*his3AI* (Figs. S4D and E, compare WT to *ste12∆*). We observed no difference in His^+^ frequency between the WT and the three M1, M3 and M6 phospho-ablative mutants in *ste12∆* or *ste12∆ cka2∆ ckb2∆* cells (Fig. 3D). These results show that the repression of Ty1 retrotransposition by CK2 is independent of the phosphorylation of the IN residues that are targeted by the kinase in vitro.

Unexpectedly, we detected a ~ 14-fold increase in p*PSP2*-Ty1-*his3AI* retrotransposition in the *ste12∆ cka2∆ ckb2∆* mutant compared to the *ste12∆* mutant, which did not depend on M1, M3 and M6 mutations in Ty1 IN (Fig. 3D). This increase is correlated with a strong increase of p*PSP2*-Ty1-*his3AI* RNA levels in the *ste12∆ cka2∆ ckb2∆* cells (Fig. S4E). This could reflect the fact that CK2 represses *PSP2* promoter activity or that CK2 destabilizes Ty1 RNAs in a post-transcriptional manner. To distinguish between these two possibilities, we measured *PSP2* mRNA levels in *cka2∆ ckb2∆* strains. In contrast to p*PSP2*-Ty1-*his3AI* mRNA levels that were strongly increased in *cka2∆ ckb2∆* mutants (Fig. S4E), we detected a slight reduction in *PSP2* mRNA levels in these mutants (Fig. S4F), indicating that CK2 does not repress the *PSP2* promoter. These data taken together suggest that CK2 might control Ty1 RNA metabolism, at (a) post-transcriptional level(s).

Collectively, these results indicate that CK2 represses Ty1 retrotransposition independently of IN amino acids phosphorylated by CK2 in vitro and without altering Ty1 integration profile. Therefore, the kinase may control Ty1 activity at other stage(s) of its replication cycle.

### Ty1 RNA, protein and cDNA levels increase in the absence of CK2

**Fig. 4:**
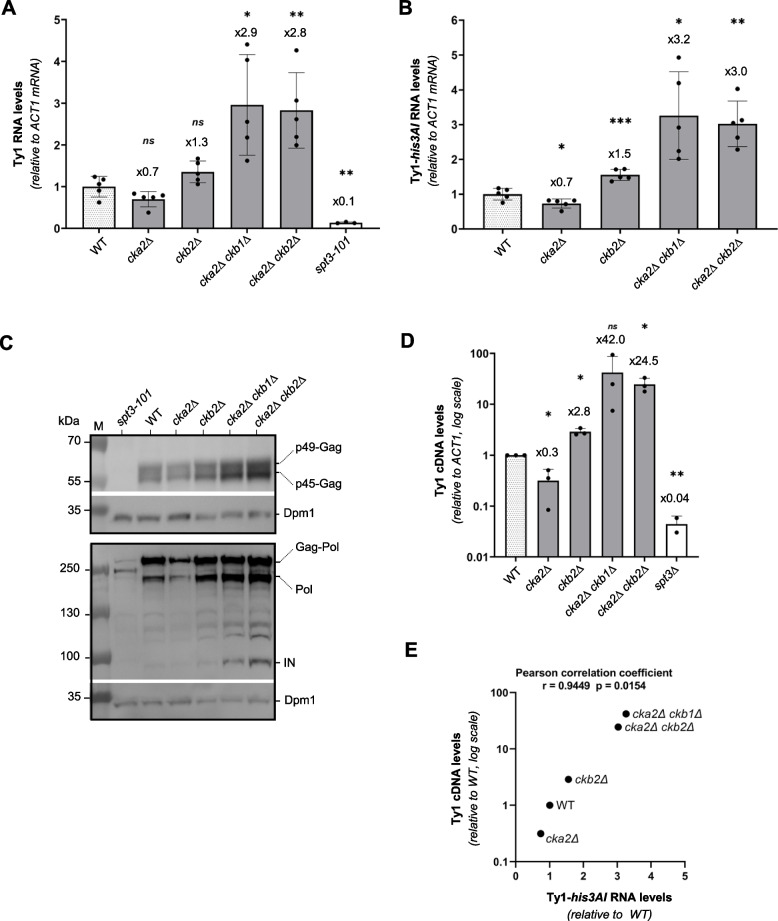
Ty1 RNA, protein and cDNA levels increase in the absence of CK2 holoenzyme. **A** Ty1 RNA levels in WT cells and different mutants of the CK2 complex, as measured by RT-qPCR (mean ± SD, *n* ≥ 3, relative to WT and normalized to *ACT1* mRNAs). **B** Ty1-*his3AI* RNA levels in WT cells and different mutants of the CK2 complex as described in panel A. **C** Whole cell protein extracts of the indicated strains analyzed by Western blot using anti-VLP antibodies revealing p49/p45-Gag proteins, and anti-IN monoclonal antibodies revealing IN, Gag-Pol and Pol intermediates. Dpm1 is a loading control. **D** Total Ty1 cDNA levels in WT cells and different mutants of the CK2 complex, as measured by qPCR (mean ± SD, *n* = 3, relative to WT and normalized to *ACT1*). **E** Total Ty1 cDNA levels (means ± SD, *n* = 3, relative to WT, values from panel A) are plotted as a function of total Ty1 RNA levels (mean ± SD, *n* ≥ 3, relative to WT, values from panel B) in the indicated strains. The Pearson correlation coefficient and associated *p*-value are indicated. The *spt3–101* or *spt3Δ* null mutants are used as controls for qPCR (Panels A and B), anti-VLP and anti-IN antibodies (Panel D) specificities because Ty1 expression is strongly decreased in these mutants. Unpaired bilateral Student’s t-test: *p < 0.05; **p < 0.01; ***p < 0.001; ****p < 0.0001

Our data suggested that CK2 controls Ty1 retrotransposition independently of its interaction with IN. In addition, the increased levels of p*PSP2*-Ty1-*his3AI* mRNA in the *cka2∆ ckb2∆* mutant (Fig. S4E) pointed to a possible control of the kinase on Ty1 RNA metabolism. To further investigate the role of CK2 in Ty1 retrotransposition, we quantified intermediates of the Ty1 replication cycle. First, we assessed Ty1 and Ty1-*his3AI* RNA levels expressed from Ty1 promoter by RT-qPCR in the different CK2 deletion mutants that impact chromosomal Ty1-*his3AI* retrotransposition frequency to varying degrees (Fig. 4A and B). Our assay revealed an approximately 3-fold increase in the level of both RNA species in the *cka2∆ ckb1∆* and *cka2∆ ckb2∆* mutants that showed the greatest increase in Ty1 retrotransposition (Fig. 4A-B, compare with Fig. 3A). Conversely, Ty1 or Ty1-*his3AI* RNA levels did not increase in the *cka2∆* mutant and slightly in the *ckb2∆* mutant, whose retrotransposition frequencies remain unchanged or moderately enhanced, respectively. Interestingly, a similar increase in Ty1-*his3AI* mRNA levels occurred in *cka2∆ ckb2∆* mutants, whether Ty1-*his3AI* was expressed from its own promoter or the *PSP2* promoter (Fig. 4B and S4E). Taken together, these data support the view that CK2 controls Ty1 mRNA levels.

Ty1 RNA plays a central role in the retrotransposition by serving as a template for both Ty1 protein translation and reverse transcription into cDNA. Therefore, the increase in Ty1 mRNA levels could explain the strong derepression of Ty1 retrotransposition in CK2 mutants. To assess the impact of Ty1 mRNA derepression on subsequent cycle steps, we analyzed Ty1 protein and cDNA levels. Anti-VLP polyclonal antibodies were used to reveal Gag proteins by western blot, while an anti-IN monoclonal antibody was used for Gag-Pol and IN detection (Fig. 4C). The levels of Gag and Gag-Pol remained unchanged in *cka2∆* or *ckb2∆* single mutants compared with WT, but slightly increased in *cka2Δ ckb1Δ* and *cka2Δ ckb2Δ* mutants. On the other hand, IN, which was barely apparent in WT cells and in the single mutants, was readily detected in *cka2Δ ckb1Δ* and *cka2Δ ckb2Δ* mutants (Fig. 4C). Thus, the increase in Ty1 protein levels occurs primarily in the *cka2Δ ckb1Δ* and *cka2Δ ckb2Δ* mutants, where the increase in Ty1 mRNA levels is highest.

We also measured the levels of unintegrated Ty1 cDNA in a subset of CK2 deletion mutants (Fig. 4D), using a qPCR methodology we developed recently [63]. A strong correlation was observed between Ty1 cDNA levels and Ty1 mRNA levels (Pearson correlation coefficient *r* = 0.945, *p* = 0,015) where a slight increase in Ty1 mRNA was associated with a strong increase in unintegrated cDNA (Fig. 4D and E). The difference in cDNA levels was also consistent with the transposition frequency measured in the various mutants (compare Fig. 4D with 3A). Specifically, the *cka2Δ ckb1Δ* and *cka2Δ ckb2Δ* mutants that displayed the highest derepression of Ty1-*his3AI* retrotransposition (Fig. 3A) also had the highest levels of Ty1 mRNA (Fig. 4A and B) and unintegrated cDNA (Fig. 4D).

In conclusion, the accumulation of Ty1 mRNA in the absence of CK2 holoenzymes could play a prominent role in the derepression of Ty1 retrotransposition, leading to increased levels of Ty1 proteins and cDNA that are essential intermediates of retrotransposition.

## Discussion

In this study, we identified many new putative partners of Ty1 integrase. We have shown that IN is phosphorylated in vivo and that the protein kinase CK2 is involved in this process. We did not find a function for this phosphorylation of IN in Ty1 retrotransposition. However, our data indicate that CK2 is a major repressor of Ty1 retrotransposition, which controls the level of cellular Ty1 RNAs*.*

Ty1 IN plays a key role in Ty1 retrotransposition as it is involved in multiple steps, from the synthesis of cDNA to its nuclear import and integration into the yeast genome. Thus, we would expect IN to have many cellular partners that regulate its functions during the replication cycle. However very few have been identified in previous studies [18, 31, 32] and in the only proteomic approach reported so far, in which IN was produced from a Ty1 element, only a dozen of IN-associated proteins were recovered, including 5 RNA Pol III subunits [38]. The fact that Ty1 IN is mostly insoluble [25, 64] may be responsible for the limited number of factors identified in this previous study. Here, by using the TChAP procedure, where ectopically expressed IN was crosslinked to its cofactors prior to purification, we recovered over 200 potential binding partners, providing a comprehensive view of the Ty1 IN interactome. The identification of IN known partners, such as Ty1 proteins and several RNA Pol I and Pol III subunits, validated our approach. Among them, several proteins, including AC40 or Ty1 reverse transcriptase had already been identified as direct partners but the TChAP cross-linking step should also allow the recovery of proteins that interact indirectly with IN. Importantly, many proteins involved in the chromatin dynamics were also retrieved, including subunits of the FACT, INO80, Paf1 and RSC complexes. Except for Paf1, where several subunits were previously found in genetic screens as Ty1 regulators [36], the proteins in the other complexes are mostly essential for cell growth and thus could not have been identified in genetic screens based on the collection of non-essential gene deletion mutants. Nevertheless, the identification of these factors, which was facilitated by the crosslink step of the TChAP procedure, was consistent with the importance of the chromatin environment for the integration process [47, 48, 65]. It has been previously shown that when the interaction with RNA Pol III is disrupted, Ty1 integration is redirected to subtelomeric regions [18, 31]. In contrast to the periodic and constraint integration upstream of Pol III-transcribed genes, Ty1 insertion in subtelomeres exhibits scattered dispersion [31], the molecular bases of which remain to be characterized. Whether (a) specific factor(s), associated with chromatin or binding to DNA, identified in the TChAP contribute(s) to Ty1 targeting at chromosome ends will require further analysis. Among them, the general regulatory factors Rap1, Reb1 and Abf1 that all bind to subtelomeric regions appear to be good candidates (Table S1) [66–68].

In this study, we focused on the interaction between Ty1 IN and the CK2 holoenzymes. Retroviral integrases undergo various post-translational modifications, such as phosphorylation, acetylation, ubiquitination and sumoylation [69], which play multiple roles in viral replication such as infectivity [19], IN interaction with cellular factors [22], IN degradation [70], cDNA integration [57, 58], or even post-integration proviral transcription [71]. In particular, IN phosphorylation has been documented to interfere with many integrase features including its stability [72] or activity [20]. In contrast, there is very little information on the phosphorylation of yeast retrotransposon integrases, with the exception of Ty5 IN. In this case, IN phosphorylation in the targeting sequence drives Ty5 integration into heterochromatin [60]. Nevertheless, mass spectrometry raw data from different proteomic approaches indicate the presence of phosphorylated Ty1 proteins in vivo [73–75]. Here, we provide several lines of evidence that Ty1 IN is phosphorylated in yeast cells and that CK2 is involved in this process. First, IN expressed ectopically alone or from a functional Ty1 element in yeast cells co-immunoprecipitated with CK2 holoenzyme (Fig. 1C and Fig. S2). Specifically, the regulatory subunit Ckb2 interacted with IN by two-hybrid (Fig. 1D). Second, IN was phosphorylated by CK2 in vitro and all modified amino acids are located in the Ckb2 binding region (Fig. 2B) which is included in the intrinsically disordered CTD [25]. Interestingly, these unstructured regions are known to be favourable for protein post-translational modifications [76]. Their phosphorylation influences protein folding, interaction with binding partners and consequently protein functions [76]. Third, IN expressed ectopically from a Ty1 element was phosphorylated in vivo and the multiple bands of lower mobility detected in a *cka2∆ ckb2∆* mutant, as compared to WT, is indicative of a role for CK2 in these modifications. The complex phosphorylation pattern of IN may reflect that the protein undergoes successive phosphorylation events during Ty1 replication. However, we could not establish whether the role of CK2 in these post-translational modifications is direct or indirect. The production of IN from a Ty1 element requires many steps taking place in different cellular compartments that could expose IN to several kinases, including CK2, during Ty1 replication. Alternatively, proteins involved in retrotransposition could promote the recruitment of kinases or a conformation favouring IN phosphorylation. Some phosphorylation could be transient and thus explain the presence of many different forms of phosphorylated INs in yeast cells. Finally, the difficulty of detecting IN phosphorylation in vivo may be related to the fact that only a fraction of the IN molecules could be modified under our experimental conditions.

Surprisingly, some CK2-dependent IN phosphorylation sites have been identified in vivo but not in vitro (Table S4B). Besides the high sensitivity of MS, this finding could be explained by a hierarchical mechanism where an initial modification by another kinase is required before the phosphorylation by CK2, a mechanism reported for several CK2 sites, which would only occur in vivo [77]. Conversely, because CK2 can control various kinase activities [78], it could indicate that one or more CK2-dependent kinases are accountable for the phosphorylation of these residues in vivo.

We did not detect any change in the frequency of Ty1 retrotransposition, when phospho-ablative mutations were introduced on all in vitro CK2-targeted IN amino acids. Moreover, there was no change in the in vitro catalytic activity of a phosphomimetic IN mutant. We also did not observe a change in the integration profile of Ty1 in CK2 mutants. These data suggest that CK2-dependent IN modification does not influence Ty1 integration, at least upon our experimental conditions. CK2 activity is regulated in distinct cellular processes or in response to cellular stress (for a review see [79]), suggesting that CK2-dependent phosphorylation of IN could be important under specific conditions known to regulate Ty1 retrotransposition, such as ionizing radiation, oxidative or nutritional stresses [80–82]. This phosphorylation could also be part of the DNA damage response since CK2 is a key player of this response [83] and Ty1 retrotransposition is stimulated transcriptionally and post-transcriptionally by DNA damage [82, 84–86]. It was reported that the interaction between the 115 C-terminal residues of Ty1 IN and RT are critical for RT polymerase activity [26, 27, 87]. Since some phosphorylated residues are located within this 115-residue region of IN, the phosphorylation of IN might affect the reverse transcription step. On the other hand, a former study showed that IN overexpression facilitates the integration of DNA fragments with no similarity to Ty1 cDNA into the yeast genome [88]. Thus, IN phosphorylation could also limit this unconventional activity of Ty1 to maintain genome integrity. Alternatively, IN post-translational modification could transiently influence Ty1 transcription right after de novo integration as reported for HIV IN [71].

In contrast to the phospho-ablative IN mutants, several CK2 mutants strongly derepressed Ty1 retrotransposition. Ty1 derepression was previously shown with a *ckb2∆* mutant [36] and we observed a similar phenotype with the deletion of the other regulatory subunit Ckb1. Since the absence of the Ckb1 or Ckb2 regulatory subunit impairs the integrity of the CK2 holoenzyme [51], and as we did not observe a synergistic effect in the absence of both Ckb1 and Ckb2, these results point to a role of CK2 holoenzymes in Ty1 regulation. In contrast, no significant changes were detected in the absence of either catalytic subunit. However, the absence of the Cka2 catalytic subunit was synergistic with that of Ckb1 or Ckb2, suggesting the presence of some unstable Cka2/Ckb subcomplexes in vivo that would repress Ty1 retrotransposition.

Our data indicate that CK2 repression acts on Ty1 RNA metabolism. The control by CK2 could occur at a post-transcriptional step because the increase in Ty1-*his3AI* mRNA is detected in CK2 mutants, even when Ty1 is expressed from the promoter of *PSP2*, unless CK2 regulates the activity of (a) Ty1 transcription factor(s) known to bind to Ty1 internal sequences still present in the pPSP2-Ty1-*his3AI* reporter [86]. Ste12 that binds to a motif in *GAG* open reading frame and activates Ty1 transcription is probably not this factor because CK2 still regulates Ty1 RNA levels in the absence of Ste12 (Fig. S4E). Even though Ty RNAs are very abundant [89], increasing the level of Ty1 RNA in CK2 has a strong effect on unintegrated cDNA levels and Ty1 retrotransposition. A similar effect has been recently described in Nup84 complex mutants. In this case, nuclear pore complexes were shown to restrict Ty1 retrotransposition by repressing Ty1 transcription [63]. Although additional experiments will be required to determine how CK2 regulates mRNA level and whether CK2 control operates on Ty1 RNA export, localization, or stability, both studies point to the importance of restricting Ty1 mRNA levels to avoid excessive Ty1 retrotransposition.

## Material & methods

### Plasmids, primers, yeast strains and growth

The *S. cerevisiae* strains used in this study are listed in Table S5 and were grown in standard yeast extract peptone dextrose (YPD) or synthetic complete (SC) media lacking appropriate amino acids. Deletions of CK2 subunits were created in strains LV1434 containing a chromosomal Ty1-*his3AI-Δ1–3114* [35], LV1689 or in the reference strain BY4741, by one-step gene replacement, using PCR fragments of *hphMX* or *kanMX* cassettes, flanked with 5′ and 3′ sequences of the deleted gene (Phusion high-fidelity DNA Polymerase, Thermo Fischer). Yeast transformations were performed by the lithium acetate procedure. All constructs and gene replacements were checked by PCR analysis. All mutations introduced in IN sequence to block phosphorylation were constructed by Bridge mutagenesis (Mehta and Singh, 1999) and validated by sequencing (Eurofins Genomics). The plasmids and primers used in this study are reported in Tables S6 and S7. The C-tag present in GAD-IN fusion is a 4-amino acid peptide tag (glutamic acid-proline-glutamic acid-alanine) [90].

### Tandem chromatin affinity purification and mass spectrometry analysis

The TChAP method was mainly performed as described in [42] with the following modifications. Yeast strain LV1689 transformed with pCM185-IN-HBH or pCM185-IN was grown overnight at 30 °C in the presence of doxycycline (Dox, 10 μg/ml), in SC medium lacking tryptophane to maintain plasmid selection. The next day, cells were diluted in fresh medium without Dox to reach the exponential phase the next morning. At OD_600_ = 1, 2 L of cells were crosslinked for 20 min with 1% formaldehyde (prepared in 1X PBS). The cells were centrifuged at 3500 rpm at 4 °C for 5 min and washed 3 times with ice cold PBS (1X) to remove all the formaldehyde, and once in sucrose buffer (300 mM sucrose, 1% Triton X-100, PBS 1X) before to be frozen at − 80 °C. The cells were then disrupted by passage through an Eaton press, thawed on ice and centrifuged at 6000 rpm at 4 °C for 6 min. Pellets were washed three times with PBS 1X containing 0,5% Tween 20 and then resuspended in 15 ml of urea buffer (20 mM NaH_2_PO_4_/Na_2_HPO_4_ pH = 7.5, 6 M Urea, 1 M NaCl, 0.1% SDS, 0.1% Sarkosyl, 10 mM Imidazole and 5 mM *β*-mercaptoethanol, pH = 8) supplemented with EDTA-free protease inhibitor mixture (Roche) and 100 mM PMSF. The chromatin was solubilized and sheared using a Q700 sonicator with a microtip probe (Qsonica). The sonicator was set to 5 cycles of 10 s ON followed by 50 s OFF with 70% amplitude. The probe and the extracts were kept cold on ice (temperature < 20 °C). The lysates were clarified by centrifugation for 30 min at 10,000 rpm at 10 °C. Lysates from 8 L of yeast treated cells were then subjected to a 5 ml Nickel affinity purification using an AKTA purifier system (GE Healthcare) equilibrated in the urea buffer. To avoid non-specific binders, the sample was first loaded onto a 5 ml sepharose fast flow column screwed to another 5 ml nickel sepharose column allowing pre-clearing of the extract before metal affinity capture. The sample was injected for a total of 3 times. Upon extensive wash (urea buffer adjusted at pH = 6.3), the proteins were recovered with the elution buffer (urea buffer adjusted at pH = 4.3) and eluted fractions were immediately neutralized to pH = 8 by the addition of Tris-HCl 1 M pH = 8.8 and incubated overnight on a rotating wheel at 10 °C with streptavidin magnetic sepharose slurry beads (GE-healthcare) equilibrated in urea buffer. The beads were washed once with urea buffer and 3 times with PBS (1X). To elute the proteins, the beads were resuspended in 500 μl of reversal buffer (250 mM Tris HCl pH = 8.8, 2% SDS and 0.5 M *β*-mercaptoethanol) and boiled at 95 °C (3 times 5 min at 95 °C followed by 30 s on ice). The final elution was transferred to a low-protein binding Eppendorf tube and the proteins were precipitated by the addition of Trichloro-acetic acid (20%) and incubated 10 min on ice. The protein pellet was then collected by centrifuging the mixture at 4 °C at 15,000 rpm for 15 min. The pellet was washed twice with 1 ml of ice-cold acetone to remove the excess TCA and dried under the hood for 5 min. Finally, the pellet was resuspended in 20 μl of reversal buffer and boiled for 5 min at 95 °C. The protein sample was then diluted with a 5X sample buffer (Thermo Fisher) according to the supplier’s protocol and resolved by SDS-PAGE on a 4–12% gradient NuPAGE (Thermo Fischer) gel. The protein bands were visualized by staining the gel with Imperial blue and de-staining with water overnight. The following day the protein bands were sliced from the gel and sent to a Mass Spectrometry Laboratory facility (IBB, Warsaw, Poland). Mass spectrometry analysis and curation of the data were performed as previously described [42].

### Protein purification and phosphorylation analysis

Recombinant IN, Inc., IN^M1^ to IN^M6^ and IN^M5D^ mutants were prepared from *E. coli* as previously described [25] from plasmids listed in Table S6. Native CK2 holoenzyme was purified from *S. cerevisiae* cells expressing TAP-tagged Ckb2 by TAP-tag affinity chromatography [91] up to tobacco etch virus (TEV) protease cleavage.

Kinase reaction (20 μl) was performed at 30 °C for 30 min in kinase buffer (100 mM NaCl, 20 mM Tris-HCl (pH = 8), 10 mM MgCl_2_, 1 mM DTT, 100 μM cold ATP and 1 μCi [γ^-32P^] ATP (6000 Ci/mmol,10 mCi/ml, Perkin Elmer) with recombinant proteins (200–500 ng) and yeast purified CK2 or recombinant human CK2 (NEB, P6010S). Proteins were resolved by SDS-PAGE and the gels were exposed to autoradiography or stained with Coomassie blue.

For analysis of in vivo phosphorylation, LV1689 cells transformed with pCM185-IN, Ty1 or M6 mutants were grown overnight at 30 °C in SC medium lacking tryptophan to maintain plasmid selection in the presence of 1 μg/ml of doxycycline. Cells were washed and diluted in fresh medium without doxycycline to reach the exponential phase (OD_600_ = 1) the day after. Total protein extracts were prepared from 10 OD_600_ of cells by TCA lysis method and analyzed by Western blot after 6% SDS-PAGE (Novex Tris-Glycine, Thermo Fisher) or 7% Zn^2+^-Phos-tag™ SDS-PAGE according to the manufacturer’s protocol (20 μM Phos-Tag™, Fujifilm Wako).

### Assays for Ty1 IN catalytic activity in vitro

Strand transfer activity of IN WT or IN^M5D^ (2 μM) was assayed using Ty1 U3 DNA sequence as substrates as previously described [25]. Briefly, INs were incubated with 20 pmol of fluorescent DNA substrates on ice in integration buffer (10 mM Tris-HCl pH 7.5, KCl 125 mM, 0.8% glycerol, 5% PEG 8000, 5 mM MgCl_2_, 0.1 mM DTT) in a 20 μl reaction volume. After 15 min at 30 °C, the reaction was stopped by the addition of 20 μl of loading buffer (20 mM Tris-HCl pH 7.5, 8 M urea, 0.5% SDS, 2X Tris-Borate-EDTA). The sample was boiled 5 min at 95 °C and loaded on a 20% polyacrylamide/8 M urea gel. After electrophoresis, the DNA was visualized using an Odyssey CLx fluorescence near-infrared imaging system at 700 nm.

### Immunoprecipitation experiments

Cells transformed with pCM185-IN-HBH or pCM185-Ty1 were grown to exponential phase at 30 °C as described above. Fifty ml of cells (OD_600_ = 1) were re-suspended in 500 μl of IP buffer (50 mM HEPES-KOH pH = 7.5, 300 mM NaCl, 1 mM EDTA, 0.05% NP40, 0.5 mM DTT, 5% glycerol, 1 mM PMSF and protease inhibitor cocktail tablet (Roche complete)). Whole-cell extracts were prepared using acid-washed glass beads (Sigma, G8772) and incubated for 2 h at 4 °C with 50 μl of IgG magnetic PanMouse Dynabeads (Thermo Fisher) equilibrated with IP buffer. The beads were washed 3 times with 1 mL of IP Buffer and the proteins eluted from the beads by adding 20 μl of sample buffer (50 mM Tris-HCl, 2% SDS, 10% glycerol, 2% *ß*-mercaptoethanol) and boiling at 95 °C for 5 min. Eluted proteins were resolved by 10% SDS-PAGE and detected by Western blot as described below.

### Two-hybrid assays

Assays were performed using the host strain Y190 (reporter genes *LacZ* and *HIS3*). Cultures were grown overnight at 30 °C in SC medium lacking leucine and tryptophan to maintain plasmid selection. Cells were plated on SC medium lacking leucine, tryptophan and histidine and containing 0.2 mM 3-Amino-1,2,4-triazole to suppress leaky *HIS3* expression in Y190 strain. Plates were incubated 2 days at 30 °C. Activation of the *LacZ* reporter gene was monitored using a X-Gal agarose overlay assay.

### Retrotransposition assays

Four independent clones of strains containing the Ty1-*his3AI* chromosomal reporter were grown to saturation for at least 24 h at 30 °C in YPD. Each culture was diluted thousand-fold in YPD and grown for 4 days to saturation at 20 °C, which is the optimal temperature for Ty1 retrotransposition. Aliquots of cultures were plated on YPD (100 μl at 10^− 5^) and SC-His (0.2 to 1.5 mL, depending on the expected frequency). Plates were incubated for 3–4 days at 30 °C and colonies were counted to determine the fraction of His^+^ prototrophs. A retrotransposition frequency was calculated as the median of the ratios of number of His^+^ cells to viable cells for each of the four independent clones. Retrotransposition frequencies were then defined as the mean of at least three medians.

### High-throughput Ty1 integration data acquisition and analysis

Libraries of de novo Ty1 integration events in WT and *cka2∆ ckb2∆* strains were prepared as described in [92]. In brief, BY4741 and yABA11 strains were transformed with pGTy1-*his3AI*-SCUF [47]. Total genomic DNA was extracted from 47,660 (for LV1434) and 63,867 (for LV1569) His^+^ colonies recovered from ten independent cultures grown at 20 °C in the presence of galactose. Fasteris prepared and sequenced Illumina libraries. Finally, 46,331 and 52,676 unique reads were analyzed for the WT and *cka2∆ ckb2∆* strains, respectively. Proper aligned paired reads were sorted as described in Asif-Laidin (2020). Ty1 de novo integrations were assigned to the corresponding genomic feature. Analyses were performed using in-house R pipelines (http://www.R-project.org), to compare the associations of integration profiles (both in vivo and in silico) with selected genomic features (subtelomeres [93], telomeres, retrotransposons, ARS and random, 1 × 10^5^ Ty1 random insertions generated in silico).

### PCR assays for detection of Ty1 integration events

After retrotransposition induction as described above, total genomic DNA was extracted from yeast cultures grown at 20 °C for 5 days by classical phenol-chloroform method [92] and double-strand DNA concentration was determined using Qubit™ Fluorometer (Thermo Fisher). 30 ng of DNA were used for PCR assays at the *SUF16 tDNA* locus or 75 ng for PCR at subtelomeric *HXT13*, *HXT15*, *HXT16* and *HXT17* loci. PCR was performed following standard protocol for Phusion High-Fidelity DNA Polymerase (Thermo Fischer) using primers hybridizing in Ty1 *POL* sequence (O-AB46) and at *SNR33*, a gene located downstream of *SUF16* (O-AB91), or at the four *HXT* genes (O-ABA27). The PCR program was the following: 2 min at 98 °C; 10 s at 98 °C, 30 s at 61 °C, 1 min at 72 °C (30 cycles); 5 min at 72 °C. PCR products were separated using a 1.5% agarose gel. PCR on *ACT1* using specific primers (O-AB18 and O-AB19) was performed as a loading control.

### RT-qPCR analysis of mRNA levels

Total RNAs were extracted from mid-log yeast cultures grown 16 h at 20 °C in YPD as described above using the Nucleospin RNA II kit (Macherey-Nagel) and were reverse transcribed with Superscript-II reverse transcriptase (Invitrogen). cDNA quantification was achieved by real-time qPCR with a QuantStudio 5 system (Thermo Fischer) using the Power Track SYBR Green Master mix (Thermo Fisher). The amounts of the RNAs of interest were normalized relative to *ACT1* mRNA values and further set to 1 for WT cells. Primers used were: all Ty1: O-AMA14/15; Ty1-*HIS3*: O-AMA34/35; *ACT1*: O-AMA10/11; pTet-IN: O-ABA131/133; *PSP2* O-AMA344/345 and are described in Table S7.

### Western blot and antibodies

To assess IN and Gag protein levels, overnight pre-cultures were diluted to OD_600_ = 0.01 and grown to mid-log at 20 °C (OD_600_ = 1–2). Total proteins were extracted from 10 OD_600_ cultures by the TCA lysis method. Samples were separated on a commercial 10% SDS-PAGE for Gag and 4–12% SDS-PAGE for IN (Bolt™, Thermo Fischer) and transferred to nitrocellulose.

Western blot analysis was performed using the following antibodies: polyclonal anti-VLP to detect p49/p45-Gag polypeptides [82] (1/10,000), monoclonal anti-integrase (8B11, a gift from J. Boeke; 1/100, in Fig. 4C) or rabbit polyclonal IN antibodies (1/1000 to 1/7500, prepared from recombinant IN), anti-TAP tag (1/10,000, Thermo Fischer, Invitrogen, CAB1001), monoclonal anti-Dpm1 (1/2000, Thermo Fischer, Invitrogen, 5C5A7), anti-Pgk1 (1/10,000, Thermo Fischer, Invitrogen, 459,250), anti-Act1 (1/10,000, Abcam, Ab8224) at 4 °C from 2 h to overnight. Signal detection was performed using the Pierce ECL Western blotting substrate (Thermo Fischer) and images were obtained using camera (Fusion FX, Lourmat) or by autoradiography.

## Supplementary Information

**Additional file 1: Figs. S1 to S4**.

**Additional file 2: Table S1.** List of the proteins identified by TChAP.

**Additional file 3: Table S2.** MS Data set of Ty1 proteins identified by TChAP.

**Additional file 4: Table S3.** Gene Ontology enrichment analysis of Biological Process and Cellular Component of Ty1 IN partners identified by TChAP.

**Additional file 5: Table S4.** Identification of IN phosphorylated residues. (**A**) Netphos 3.1 server-prediction results of Ty1 IN. (**B**) Identification of IN phosphorylated peptides by LC MS-MS.

**Additional file 6: Table S5-S7.** Yeast Strains, plasmids and primers used in this study.

## Data Availability

The mass spectrometry proteomics data have been deposited to the ProteomeXchange Consortium via the PRIDE partner repository with the dataset identifier PXD027420 and 10.6019/PXD027420. Ty1 de novo insertion data is deposited to Sequence Read Archive under accession number PRJNA821248.
