# Supplementary material for: A proteomic screen of Ty1 integrase partners identifies the protein kinase CK2 as a regulator of Ty1 retrotransposition
Source: Mob DNA. 2022 Nov 18;13:26. doi: 10.1186/s13100-022-00284-0 (PMC9673352; doi:10.1186/s13100-022-00284-0)
Supplement: Supplementary file 1 — Additional file 1: Figs. S1 to S4. [file 13100_2022_284_MOESM1_ESM.pdf]

Additional file 1 of a proteomic screen of Ty1 integrase partners identifies the protein kinase CK2 as a regulator of Ty1 retrotransposition

A

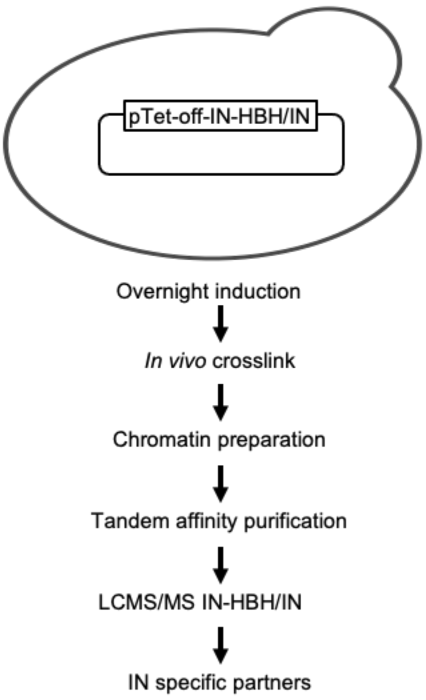

B

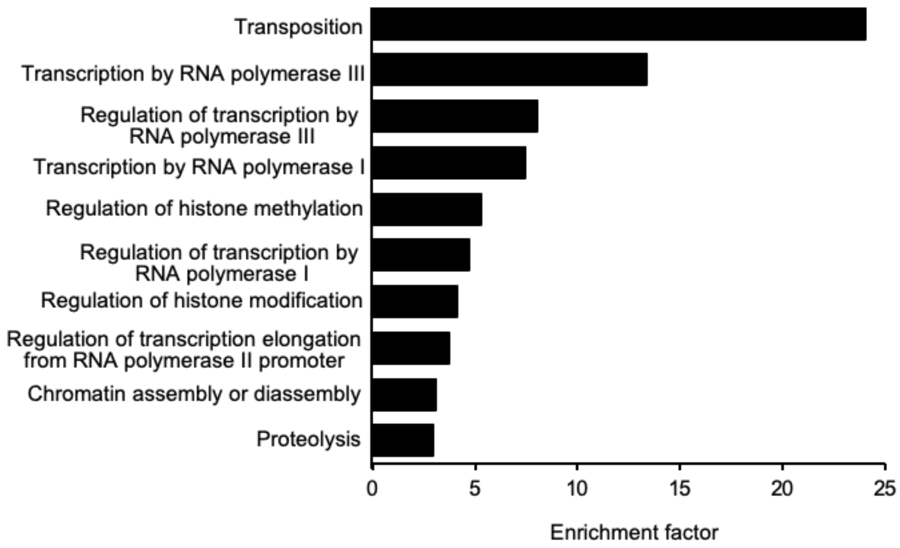

Figure S1.

(A) Overview of the TChAP procedure. Yeast cells transformed by either pTet-off-IN or pTet-off-IN-HBH were diluted in SC medium in the absence of doxycycline to allow the induction of IN proteins and grown overnight to reach the exponential phase the next day. Cells were cross-linked, lysed and sonicated chromatin was affinity-purified over consecutive nickel and streptavidin resins before protein identification by MS.

(B) Functional classification of IN partners based on Gene Ontology (GO) biological process. GOrilla algorithm (89) was used to retrieve statistically significant enriched GO terms within the complete set of proteins identified using the TChAP procedure. Numbers on X-axis indicate the enrichment in each identified GO cluster. Full list of the enriched GO terms is available in Table S3.

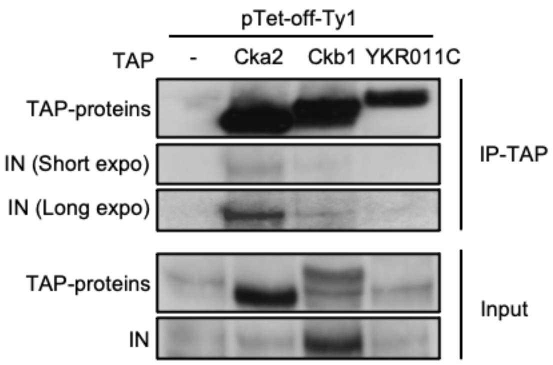

Figure S2.

CK2 is associated with IN expressed from a Ty1 element *in vivo*. Yeast whole cell extracts prepared from WT (-) or strains expressing TAP-tagged CK2 subunits transformed by pTet-off-Ty1 were immunoprecipitated with IgG beads. Proteins were analyzed by Western blotting using anti-IN or anti-TAP antibodies. TAP-YKR011C is used as a negative control.

A

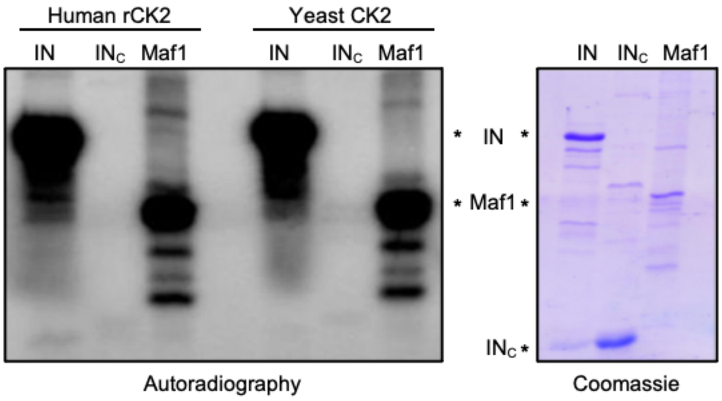

B

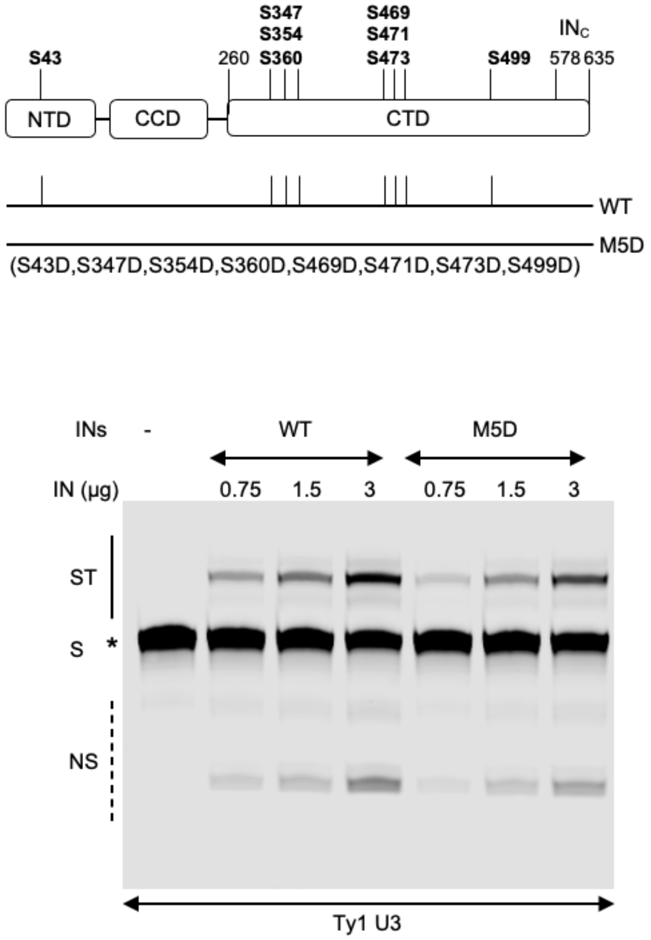

Figure S3.

(A) Full-length IN (200 ng), IN_c (500 ng) or Maf1 (200 ng) were subjected to *in vitro* radioactive phosphorylation assays with commercial recombinant human CK2 or purified yeast CK2 holoenzyme as indicated. Incorporation of $\gamma^{32}P$ is detected by

autoradiography (left panel), the loading of the recombinant proteins is analyzed by Coomassie blue staining (right panel). Full length IN, IN_c and Maf1 are indicated (*).

(B) Strand transfer (ST) and non-specific endonuclease (NS) activities of recombinant WT IN and the phosphomimetic mutant INM5D (the eight serines residues (S) phosphorylated by CK2 *in vitro* were mutated to aspartic acid (D)) were performed in an oligonucleotide integrase assay as described (2) using 30 bp fluorescent double-stranded DNA probes mimicking Ty1 LTR-U3 (Ty1 U3). Samples were loaded on a 20% denaturing polyacrylamide gel that was then analyzed using an Odyssey CLx imaging system. Dark lines indicate the positions of ST products, dashed lines the NS products (and the shortmers) and asterisks the substrates (S).

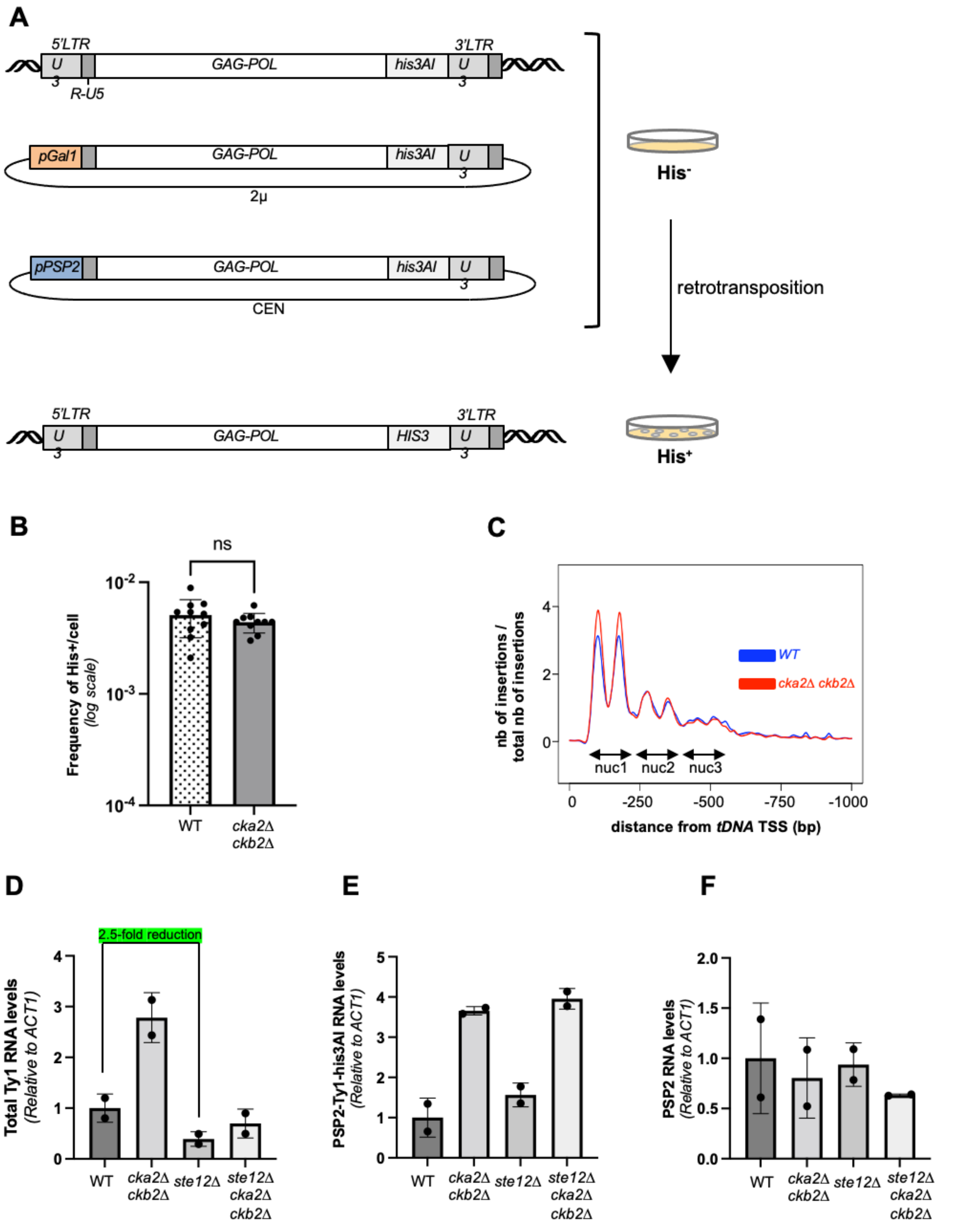

Figure S4.

(A) Schematic of p*GAL1*-Ty1-*his3AI* and p*PSP2*-Ty1-*his3AI* relative to a chromosomal Ty1-*his3AI* element with the standard LTR promoter. The p*GAL1*-Ty1-*his3AI* and p*PSP2*-Ty1-*his3AI* reporters have a *GAL1* or a *PSP2* promoter in place of the U3 region of Ty1 promoter, while retaining the Ty1 transcription start site and R-U5 region of the 5'LTR. The principle of Ty1-*his3AI* retrotransposition assay is depicted on the

right side. An intron is inserted in the *HIS3* gene in an antisense orientation in a spliceable orientation in the Ty1 transcript resulting in a Ty1 complementary DNA (cDNA) bearing a functional *HIS3* gene. Upon Ty1-HIS3 cDNA integration into the host genome, cells give rise to His+ colonies.

(B) Retrotransposition frequencies in WT and *cka2*Δ *ckb2*Δ cells (log scale, mean±SD of eight independent cultures/condition) of a p*GAL1*-Ty1-*his3AI* reporter carried on a multicopy plasmid. Same cultures as those used for *de novo* Ty1-*HIS3* integration event sequencing. ns, not significant, Welch's t-test with comparison to the WT strain.

(C) Ty1 insertion profile upstream of *tDNA*s. Total genomic DNA extracted from WT and *cka2*Δ *ckb2*Δ cell cultures was prepared for Ty1 *de novo* integration event sequencing (87). Ty1 insertions are computed in a 1 kb window upstream of all the 275 nuclear *tDNA*s (position 0 in the graph). Each position is divided by the number of insertions at this position (weight). The Smoothing curves indicate the general trend.

(**D**) Total Ty1 RNA levels in WT, *cka2Δ ckb2Δ*, *ste12*Δ and *ste12*Δ *cka2Δ ckb2Δ* cells, as measured by RT-qPCR (mean±SD, n=2, relative to WT and normalized to *ACT1* mRNAs).

(**E**) Ty1-*his3AI* RNA levels expressed from a p*PSP2*-Ty1-*his3AI* reporter carried on a centromeric plasmid in WT, *cka2Δ ckb2Δ*, *ste12*Δ and *ste12*Δ *cka2Δ ckb2Δ* cells, as measured by RT-qPCR (mean±SD, n=2, relative to WT and normalized to *ACT1* mRNAs).

(**F**) *PSP2* RNA levels in WT, *cka2Δ ckb2Δ*, *ste12*Δ and *ste12*Δ *cka2Δ ckb2Δ* cells, as measured by RT-qPCR (mean±SD, n=2, relative to WT and normalized to *ACT1* mRNAs).

Supplemental information

Additional file 1: Supplementary figure S1-S4

Additional file 2: Table S1. List of the proteins identified by TChAP.

Additional file 3: Table S2. MS Data set of Ty1 proteins identified by TChAP.

Additional file 4: Table S3. Gene Ontology enrichment analysis of Biological Process and Cellular Component of Ty1 IN partners identified by TChAP.

Additional file 5: Table S4. Identification of IN phosphorylated residues.
(A) Netphos 3.1 server-prediction results of Ty1 IN.
(B) Identification of IN phosphorylated peptides by LC MS-MS.

Additional file 6: Table S5-S7. Yeast Strains, Plasmids and Primers used in this study
