# Supplementary material for: A proteomic screen of Ty1 integrase partners identifies the protein kinase CK2 as a regulator of Ty1 retrotransposition
Source: Mob DNA. 2022 Nov 18;13:26. doi: 10.1186/s13100-022-00284-0 (PMC9673352; doi:10.1186/s13100-022-00284-0)
Supplement: Supplementary file 6 — Additional file 6: Table S5-S7. Yeast Strains, plasmids and primers used in this study. [file 13100_2022_284_MOESM6_ESM.pdf]

Supplementary Table S5 - Yeast Strains used in this study

Strain code	Name	Genotype	Origin
yABA19	WT	MATa *his3Δ1 leu2Δ0 met15Δ0 ura3Δ0 trp1Δ::NatMX*	This study
yABA21	*cka2Δ ckb2Δ*	MATa *his3Δ1 leu2Δ0 met15Δ0 ura3Δ0 trp1Δ::NatMX cka2Δ::HphMX ckb2Δ::KanMX*	This study[d,g]
LV1434	WT	MATα Ty1-*his3AI*-*Δ*1-3114 *his3-Δ1 leu2-Δ0 lys2-Δ0 ura3Δ0*	(1)
yABA15	Ty1-*his3AI cka1Δ*	MATα Ty1-*his3AI*-*Δ*1-3114 *his3-Δ1 leu2-Δ0 lys2-Δ0 ura3Δ0 cka1Δ::KanMX*	This study[a]
yABA16	Ty1-*his3AI cka2Δ*	MATα Ty1-*his3AI*-*Δ*1-3114 *his3-Δ1 leu2-Δ0 lys2-Δ0 ura3Δ0 cka2Δ::KanMX*	This study[b]
yABA17	Ty1-*his3AI ckb1Δ*	MATα Ty1-*his3AI*-*Δ*1-3114 *his3-Δ1 leu2-Δ0 lys2-Δ0 ura3Δ0 ckb1Δ::KanMX*	This study[c]
yABA18	Ty1-*his3AI ckb2Δ*	MATα Ty1-*his3AI*-*Δ*1-3114 *his3-Δ1 leu2-Δ0 lys2-Δ0 ura3Δ0 ckb2Δ::KanMX*	This study[d]
yABA24	Ty1-*his3AI cka1Δ ckb1Δ*	MATα Ty1-*his3AI*-*Δ*1-3114 *his3-Δ1 leu2-Δ0 lys2-Δ0 ura3Δ0 cka1Δ::KanMX ckb1Δ::HphMX*	This study[a,e]
yABA25	Ty1-*his3AI cka1Δ ckb2Δ*	MATα Ty1-*his3AI*-*Δ*1-3114 *his3-Δ1 leu2-Δ0 lys2-Δ0 ura3Δ0 cka2Δ::KanMX ckb2Δ::HphMX*	This study[b,f]
yABA26	Ty1-*his3AI cka2Δ ckb1Δ*	MATα Ty1-*his3AI*-*Δ*1-3114 *his3-Δ1 leu2-Δ0 lys2-Δ0 ura3Δ0 cka2Δ::HphMX ckb1Δ::KanMX*	This study[c,g]
yABA27	Ty1-*his3AI ckb2Δ ckb1Δ*	MATα Ty1-*his3AI*-*Δ*1-3114 *his3-Δ1 leu2-Δ0 lys2-Δ0 ura3Δ0 ckb2Δ::KanMX ckb1Δ::HphMX*	This study[d,e]
yABA28	Ty1-*his3AI cka2Δ ckb2Δ*	MATα Ty1-*his3AI*-*Δ*1-3114 *his3-Δ1 leu2-Δ0 lys2-Δ0 ura3Δ0 cka2Δ::HphMX ckb2Δ::KanMX*	This study[d,g]
BY4741	WT	MATa *his3Δ1 leu2Δ0 met15Δ0 ura3Δ0*	Euroscarf
yABA11	*cka2Δ ckb2Δ*	MATa *his3Δ1 leu2Δ0 met15Δ0 ura3Δ0 cka2Δ::HphMX ckb2Δ::KanMX*	This study[d,g]
yABA35	*ste12Δ*	MATa *his3Δ1 leu2Δ0 met15Δ0 ura3Δ0 ste12Δ::LEU2*	This study
yABA37	*ste12Δ cka2Δ ckb2Δ*	MATa *his3Δ1 leu2Δ0 met15Δ0 ura3Δ0 cka2Δ::HphMX ckb2Δ::KanMX ste12Δ::LEU2*	This study[d,g,h]
LV1757	Ty1-*his3AI spt3Δ*	MATα Ty1-*his3AI*-*Δ*1-3114 *his3-Δ1 leu2-Δ0 lys2-Δ0 ura3Δ0 spt3Δ::HphMX*	(2)
LV47	*spt3-101*	MATα *ura3Δ851 trp1Δ63 his3Δ200 spt3-101*	
ByΔste12	*ste12Δ*	MATalpha *his3Δ1 leu2Δ0 trp1Δ met15Δ0 ste12Δ::URA3*	This study
dCKΔste12	*ste12Δ cka2Δ ckb2Δ*	MATalpha *his3Δ1 leu2Δ0 trp1Δ met15Δ0 ste12Δ::URA3 cka2Δ::HphMX ckb2Δ::KanMX*	This study[d,g]
Y190		MATa, *ura3-52*, *his3-Δ200*, *lys2-801*, *ade2-101*, *trp1-901*, *leu2-3,112*, *gal4Δ*, *gal80Δ*, *URA3::GAL1-lacZ*, *LYS2::GAL4(UAS)-HIS3,*cyh^R	(3)
TAP-tagged strains	SC1131 (TAP-Cka1)<br>SC6163 (TAP-Cka2)<br>SC1987 (TAP-Ckb1)<br>SC1485 (TAP-Ckb2)<br>SC1870 (TAP-Tup1)<br>SC5005 (TAP-YKR011C)		Yeast TAP-tagged library, Cellzome

a. *CKA1* complete CDS was deleted by a KanMX cassette amplified from the gDNA of strain BY4741 *cka1Δ::KanMX* from Euroscarf strain collection clone Id:Y01428

b. *CKA2* complete CDS was deleted by a KanMX cassette amplified from the gDNA of BY4741 *cka2Δ::KanMX* from Euroscarf strain collection clone Id:Y01837

c. *CKB1* complete CDS was deleted by a KanMX cassette amplified from the gDNA of BY4741 *ckb1Δ::KanMX* from Euroscarf strain collection clone Id: Y04387

d. *CKB2* complete CDS was deleted by a KanMX cassette amplified from the gDNA of BY4741 *ckb1Δ::KanMX* from Euroscarf strain collection clone Id: Y01815

e. *CKB1* complete CDS was deleted by a HphMX cassette amplified from pAG32.

f. *CKB2* complete CDS was deleted by a HphMX cassette amplified from pAG32.

g. *CKA2* complete CDS was deleted by a HphMX cassette amplified from pAG32.

Supplementary Table S6 - Plasmids used in this study

Name	Description	Origin
pAG32	for deletion	(4)
pRS305	for deletion	
pFA6a-HphMX6	for deletion	(5)
p*GAL*-Ty1	2μ *AmpR URA3* p*GAL1*-Ty1-*his3AI*	(6)
pABA19	CEN *AmpR LEU2* p*PSP2*-Ty1-*his3AI*	J. Curcio
pABA20	CEN *AmpR URA3* p*PSP2*-Ty1-*his3AI*	This study
p*PSP2*-Ty1^{M1}	CEN *AmpR URA3* p*PSP2*-Ty1^{M1}- (IN mutations $S_{469}A$, $S_{471}A$, $Y_{472}F$, $S_{473}A$, $T_{477}A$, $T_{480}A$, $S_{499}A$)	This study
p*PSP2*-Ty1^{M3}	CEN *AmpR URA3* p*PSP2*-Ty1^{M3}-*his3AI* (IN mutations $S_{347}A$, $S_{354}A$, $S_{360}A$, $S_{411}A$, $S_{469}A$, $S_{471}A$, $Y_{472}F$, $S_{473}A$, $T_{477}A$, $T_{480}A$, $S_{499}A$)	This study
p*PSP2*-Ty1^{M6}	CEN *AmpR URA3* p*PSP2*-Ty1^{M6}-*his3AI* (IN mutations $S_{43}A$, $S_{347}A$, $S_{354}A$, $S_{360}A$, $S_{411}A$, $S_{469}A$, $S_{471}A$, $Y_{472}F$, $S_{473}A$, $T_{477}A$, $T_{480}A$, $S_{499}A$)	This study
pCM185-IN-HBH	CEN *AmpR TRP1 TetO$_7$-CYC1p*-IN-HBH	(7)
pCM185-IN	CEN *AmpR TRP1 TetO$_7$-CYC1p*-IN	This study
pCM185-IN^{M6}	CEN *AmpR TRP1 TetO$_7$-CYC1p*-IN^{M6} (IN mutations $S_{43}A$, $S_{347}A$, $S_{354}A$, $S_{360}A$, $S_{411}A$, $S_{469}A$, $S_{471}A$, $Y_{472}F$, $S_{473}A$, $T_{477}A$, $T_{480}A$, $S_{499}A$)	This study
pCM185-Ty1	CEN *AmpR TRP1 TetO$_7$-CYC1p*-Ty1	This study
pCM185-Ty1^{M6}	CEN *AmpR TRP1 TetO$_7$-CYC1p*-Ty1^{M6} (IN mutations $S^{43}A$, $S^{347}A$, $S^{354}A$, $S^{360}A$, $S^{411}A$, $S^{469}A$, $S^{471}A$, $Y^{472}F$, $S^{473}A$, $T^{477}A$, $T^{480}A$, $S^{499}A$)	This study
pAS2-IN-C-tag	2μ *AmpR TRP1* GBD-IN-C-tag	This study
pAS2-Cka1	2μ *AmpR TRP1* GBD-Cka1	This study
pAS2-Cka2	2μ *AmpR TRP1* GBD-Cka2	This study
pAS2-Ckb1	2μ *AmpR TRP1* GBD-Ckb1	This study
pAS2-Ckb2	2μ *AmpR TRP1* GBD-Ckb2	This study
pACTII-IN-C-tag	2μ *AmpR LEU2* GAD-IN-C-tag	This study
pPL121/pACTII-IN	2μ *AmpR LEU2* GAD-IN	(8)
pAT26/ pACTII-IN$_{-578}$	2μ *AmpR LEU2* GAD-IN$_{-578}$	(8)
pABA10/ pACTII-IN$_{386-511}$	2μ *AmpR LEU2* GAD-IN$_{386-511}$	This study
pACTII-Cka1	2μ *AmpR LEU2* GAD-Cka1	This study
pACTII-Cka2	2μ *AmpR LEU2* GAD-Cka2	This study
pACTII-Ckb1	2μ *AmpR LEU2* GAD-Ckb1	This study
pACTII-Ckb2	2μ *AmpR LEU2* GAD-Ckb2	This study
pET17b-6H-Fh8-IN-C-tag	pBR322 *AmpR T7p*-6his-Fh8-IN optimized codon-C-tag	(2)
pET17b-6H-Fh8-IN$_c$-C-tag	pBR322 *AmpR T7p*-6his-Fh8-IN$_{578-635}$ optimized codon-C-tag	This study
pET17b-6H-Fh8-IN^{M1}-C-tag	pBR322 *AmpR T7p*-6his-Fh8IN^{M1} optimized codon-C-tag (IN mutations $S_{469}A$, $S_{471}A$, $Y_{472}F$, $S_{473}A$, $T_{477}A$, $T_{480}A$, $S_{499}A$)	This study
pET17b-6H-Fh8-IN^{M2}-C-tag	pBR322 *AmpR T7p*-6his-Fh8IN^{M2} optimized codon-C-tag (IN mutations $S_{43}A$, $S_{347}A$, $S_{354}A$, $S_{360}A$)	This study
pET17b-6H-Fh8-IN^{M3}-C-tag	pBR322 *AmpR T7p*-6his-Fh8IN^{M3} optimized codon-C-tag (IN mutations $S_{347}A$, $S_{354}A$, $S_{360}A$, $S_{411}A$, $S_{469}A$, $S_{471}A$, $Y_{472}F$, $S_{473}A$, $T_{477}A$, $T_{480}A$, $S_{499}A$)	This study
pET17b-6H-Fh8-IN^{M4}-C-tag	pBR322 *AmpR T7p*-6his-Fh8IN^{M4} optimized codon-C-tag (IN mutations $S_{43}A$, $S_{347}A$, $S_{354}A$, $S_{360}A$, , $S_{469}A$, $S_{471}A$, $S_{473}A$)	This study

pET17b-6H-Fh8-IN^{M5}-C-tag	pBR322 *AmpR T7p*-6his-Fh8IN^{M5} optimized codon-C-tag (IN mutations S_{43}A, S_{347}A, S_{354}A, S_{360}A, S_{469}A, S_{471}A, S_{473}A, S_{499}A)	This study
pET17b-6H-Fh8-IN^{M6}-C-tag	pBR322 *AmpR T7p*-6his-Fh8IN^{M6} optimized codon-C-tag (IN mutations S_{43}A, S_{347}A, S_{354}A, S_{360}A, S_{411}A, S_{469}A, S_{471}A, Y_{472}F, S_{473}A, T_{477}A, T_{480}A, S_{499}A)	This study
pET17b-6H-Sso7dIN-C-tag	pBR322 *AmpR T7p*-6his-Sso7d-IN optimized codon-C-tag optimized codon-C-tag	(2)
pET17b-6H-Sso7dIN^{M5}-C-tag	pBR322 *AmpR T7p*-6his-Sso7d IN^{M5D} optimized codon-C-tag (IN mutations S_{43}D, S_{347}D, S_{354}D, S_{360}D, S_{469}D, S_{471}D, S_{473}D, S_{499}D)	This study

pAB19 is a derivative of pBJC1280 given by Joan Curcio (CEN *AmpR LEU2* p*PSP2*-Ty1-*his3AI*) in which *LEU2* has been replaced by *URA3*.

<u>Supplementary Table S7</u> - Primers used in this study

Name	Sequence
O-AB18_Act1F	TCGTGCTGTCTTCCCATC
O-AB19_Act1R	AAACGGCTTGGATGGAAACG
O-AB46_TYBOUT	GTGATGACAAAACCTCTTCCG
O-AB91_SNR33OUT	TTTTAGAGTGACACCATCGTAC
O-ABA27_HXT (HXT17, HXT16, HXT15, HXT13)	GACATGGGCCCCTGTTGCT TATATTGT
O-AMA14_Ty1-5'2F	TGGAACGCCTCTGAGCACTC
O-AMA15_Ty1-5'2R	CATTAGGTGAGGTTAACATTG
O-AMA34-HIS3-5R	GGCGCAAATCCTGATCCAAA
O-AMA35- HIS3-5F	ACGACCATCACACCACTGAA
O-AMA10_Act1F	ACGTTACCCAATTGAACACG
O-AMA11_Act1R	AGAACAGGGTGTTCTTCTGG
O-AMA158_Adapteur F	GTAATACGACTCACTATAGGGCACGCGTGGTCGACGGCCC GGGCTGGT
O-AMA159_Adapteur R	ACCAGCCC
O-AMA344_PSP2-5F	AGCTTGACTCACCAGCTCTT
O-AMA345_PSP2-3R	TATCGTCGCCACCTTTCTCA

1. Mou Z, Kenny AE, Curcio MJ. Hos2 and Set3 promote integration of Ty1 retrotransposons at tRNA genes in Saccharomyces cerevisiae. Genetics. 2006;172(4):2157-67.
2. Nguyen PQ, Conesa C, Rabut E, Bragagnolo G, Gouzerh C, Fernandez-Tornero C, et al. Ty1 integrase is composed of an active N-terminal domain and a large disordered C-terminal module dispensable for its activity in vitro. J Biol Chem. 2021:101093.
3. Harper JW, Adami GR, Wei N, Keyomarsi K, Elledge SJ. The p21 Cdk-interacting protein Cip1 is a potent inhibitor of G1 cyclin-dependent kinases. Cell. 1993;75(4):805-16.

4. Goldstein AL, McCusker JH. Three new dominant drug resistance cassettes for gene disruption in Saccharomyces cerevisiae. Yeast. 1999;15(14):1541-53.
5. Hentges P, Van Driessche B, Tafforeau L, Vandenhaute J, Carr AM. Three novel antibiotic marker cassettes for gene disruption and marker switching in Schizosaccharomyces pombe. Yeast. 2005;22(13):1013-9.
6. Curcio MJ, Garfinkel DJ. Single-step selection for Ty1 element retrotransposition. Proc Natl Acad Sci U S A. 1991;88(3):936-40.
7. Asif-Laidin A, Conesa C, Bonnet A, Grison C, Adhya I, Menouni R, et al. A small targeting domain in Ty1 integrase is sufficient to direct retrotransposon integration upstream of tRNA genes. EMBO J. 2020;39(17):e104337.
8. Bridier-Nahmias A, Tchalikian-Cosson A, Baller JA, Menouni R, Fayol H, Flores A, et al. Retrotransposons. An RNA polymerase III subunit determines sites of retrotransposon integration. Science. 2015;348(6234):585-8.
